# From Diabetes Care to Diabetes Cure—The Integration of Systems Biology, eHealth, and Behavioral Change

**DOI:** 10.3389/fendo.2017.00381

**Published:** 2018-01-22

**Authors:** Ben van Ommen, Suzan Wopereis, Pepijn van Empelen, Hilde M. van Keulen, Wilma Otten, Marise Kasteleyn, Johanna J. W. Molema, Iris M. de Hoogh, Niels H. Chavannes, Mattijs E. Numans, Andrea W. M. Evers, Hanno Pijl

**Affiliations:** ^1^Netherlands Organization for Applied Scientific Research (TNO), Department of Microbiology and Systems Biology, Leiden, Netherlands; ^2^Netherlands Organization for Applied Scientific Research (TNO), Department of Child Health, Leiden, Netherlands; ^3^Leiden University Medical Center (LUMC), Department of Public Health and Primary Care, Leiden, Netherlands; ^4^Netherlands Organization for Applied Scientific Research (TNO), Department of Work Health Technology, Leiden, Netherlands; ^5^Department of Health, Medical and Neuropsychology, Leiden University Medical Centre, Leiden University, Leiden, Netherlands; ^6^Department of Psychiatry, Leiden University Medical Centre, Leiden University, Leiden, Netherlands; ^7^Leiden University Medical Center (LUMC), Department of Internal Medicine, Leiden, Netherlands

**Keywords:** type 2 diabetes, lifestyle, cure, nutrition, ehealth, reversible, system

## Abstract

From a biological view, most of the processes involved in insulin resistance, which drives the pathobiology of type 2 diabetes, are reversible. This theoretically makes the disease reversible and curable by changing dietary habits and physical activity, particularly when adopted early in the disease process. Yet, this is not fully implemented and exploited in health care due to numerous obstacles. This article reviews the state of the art in all areas involved in a diabetes cure-focused therapy and discusses the scientific and technological advancements that need to be integrated into a systems approach sustainable lifestyle-based healthcare system and economy. The implementation of lifestyle as cure necessitates personalized and sustained lifestyle adaptations, which can only be established by a systems approach, including all relevant aspects (personalized diagnosis and diet, physical activity and stress management, self-empowerment, motivation, participation and health literacy, all facilitated by blended care and ehealth). Introduction of such a systems approach in type 2 diabetes therapy not only requires a concerted action of many stakeholders but also a change in healthcare economy, with new winners and losers. A “call for action” is put forward to actually initiate this transition. The solution provided for type 2 diabetes is translatable to other lifestyle-related disorders.

## Introduction

Current health care in the area of lifestyle-related diseases does not focus on reversal of the cause of the disease, but rather on controlling disease corollaries by manipulating biochemical pathways (gluconeogenesis by metformin, hepatic cholesterol synthesis by statins, insulin secretion by sulfonylureas, fatty acid housekeeping by PPAR agonists, etc.). A large repertoire of tools, technologies, and medicinal treatments has been developed for this purpose. Chronic disease care and (cardiovascular) risk management have been vastly improved thanks to these possibilities. However, we are running into the situation that disease care soon becomes too costly, with a number of stakeholders that either financially profit from the *status quo* or find the effort to change it too complicated. Also, unbeneficial drivers in our healthcare economy maintain this situation, as only reductionist solutions can be patented. In the context of our current healthcare system, citizens become patients in the literal sense of the word: patiently undergoing treatments instead of playing an active role in their own health care. In the end, this is an inefficient approach for treatment of the so-called “lifestyle related diseases,” including metabolic syndrome, obesity, type 2 diabetes, and cardiovascular disease (1, 2). Moreover, we now know that our lifestyle partakes in the pathogenesis of many other diseases [e.g., inflammatory diseases like rheumatoid arthritis, COPD, gastroesophageal reflux disease, osteoarthritis, neurological diseases like Alzheimer and multiple sclerosis, and specific cancers (3–5)]. Over the past 10 years, an integrated view on health and health care was developed, embracing health as a system (i.e., including systems biology concepts and technologies), the development of disease from health as a continuum and exploiting these assets toward “P4-medicine” (Predictive, Personalized, Preventive, and Participatory) (6, 7). The personalized aspect emerged from the possibilities to quantify the causal mechanisms involved disease predisposition (genetics) and development (environment), while the participatory aspect related both to the health and medical data ownership (8) and the need for patient citizens to take optimal control of all aspects of their own health, spanning all biopsychosocial aspects (9, 10). In this article, we will focus and elaborate on type 2 diabetes as an exemplary prototype of a lifestyle-related disease, but very similar concepts and approaches are valid for many other diseases. The theoretical framework of P4 medicine and P4 health is now solid, but yet difficult to translate into daily practice of health care for a number of reasons, mostly related to conflicting stakeholder interest and cost of implementation. Some examples are emerging, but mostly in an experimental and costly setting (7, 11). Type 2 diabetes is also interesting as the disease is not only part of a continuum from health to comorbidities and preventable but also to a large extend reversible and curable with relatively simple means, once P4 or P6 health is implemented, as will be demonstrated below.

Type 2 diabetes is a “genotype–environment interaction disease,” where the diabetic phenotype is expressed as a result of accumulated environmental pressures (wrong diet, too little physical exercise, disrupted sleep, and too much stress) in concert with genes that render individuals susceptible to the disease. Over the past 50 years or so, our environment has changed in a way that has increased the burden of all four components mentioned. Reversal and cure of type 2 diabetes thus needs to focus on (1) biological reversal (i.e., using lifestyle as medicine), (2) on coping with the environmental pressures (i.e., behavioral change), and (3) on reduction of the environmental pressures (i.e., socioeconomic changes). All of these three areas will be discussed.

Thus, the healthcare approach toward lifestyle-related diseases needs to change. Huge health and economic profits can be achieved if everyone would adopt an “optimal” lifestyle. This article presents abundant scientific evidence that major reductions in obesity, type 2 diabetes, and cardiovascular disease can be achieved through lifestyle interventions (12, 13). In fact, the (economic) benefits of a lifestyle-based therapy for type 2 diabetes have been demonstrated in a 10-year study (14). Yet, the introduction of a new healthcare system for lifestyle-related diseases (both therapy and prevention) does not materialize for a number of reasons, as discussed in this article. One of the major motives for not using lifestyle measures in clinical practice is the difficulty to sustain the changes. In the short term, or in a rigidly imposed, lifestyle change can be achieved, but in long-term daily life changes easily fade away due to the lack of support and the many counteracting stimuli from environmental pressure. Other major reasons for failure of lifestyle as medicine are the lack of economic benefits in the context of the current healthcare model; and the failure to use a systems approach instead of reductionist changes (15). Thus, theory and practice differ and we face a multifactorial challenge, requiring the removal of economic, social, psychological, and biological barriers.

A reorientation of health research and care is needed, starting with (re)defining health and its underlying mechanisms, realizing that integrated participatory and personalized health optimization strategies are needed, redesigning the methods to quantify health toward the development of a new generation of health biomarkers (16), lifestyle interventions (17), supporting tools and economical values, all aiming at self-empowerment, as listed below.

1. Refocusing on flexibility as core characteristic of physiology, allowing reversibility of disease.
2. Diagnosis has to quantify much more than the medical condition. A 360° diagnosis is needed that determines all relevant biological, sociological, psychological, and contextual conditions of the patient and the trajectory toward disease need to be identified and quantified, to empower the individual to achieve a sustainable and perceivable lifestyle change.
3. Interventions will need to span all relevant bio-socio-psycho-economic factors and thus change from reductionist to systemic and from generic to fine-tuning toward personal goals.
4. Motivational tools are required in the form of personal coaching as well as ICT support.
5. Health literacy needs to be improved as part of personal health empowerment.
6. Personal health data handling needs to completely refocus by empowering the citizen/patient valorize their health data for personal health and research.
7. Together, this needs to lay the foundations of both a new approach in lifestyle-related health science and health economy.

Each of these aspects is further detailed in the paragraphs below.

## The Biology

### Type 2 Diabetes Can Be Cured

Although the definition of “cure,” “reversal,” or “remission” of type 2 diabetes has been a matter of debate (18), various lines of evidence have demonstrated that T2D is a reversible disease. Bariatric surgery generally leads to remission of type 2 diabetes in obese people, although with large heterogeneity among patients (19, 20). The mechanisms are not completely understood, but beta-cell function recovery seems to be limited (21). Lifestyle intervention can bring about significant improvements in risk factors for cardiovascular disease in T2D patients, as indicated by a meta-analysis of studies (22). Most of these studies evaluated relatively “mild” monodisciplinary interventions (i.e., only dietary advice, or education, physical activity, general advice, etc.). These are discussed in detail below. Intensive lifestyle intervention studies have shown very promising results. Reversal of type 2 diabetes by omitting sugars and starches from the diet may reduce or even abolish the need for glucose control medication. Yet, from a mechanistic point of view, cure would only be achieved if low-grade inflammation and oxidative damage are reversed, insulin sensitivity has been restored in all relevant organs, and insulin production by the beta-cell is sufficient. Organ insulin sensitivity can be restored by various lifestyle interventions, primarily through weight loss and reduction of intra-organ adipose deposits. This is discussed in the succeeding paragraphs. Improvement of beta-cell function by lifestyle intervention was described by Taylor (23). Recent mechanistic evidence documenting diet-induced beta-cell regeneration adds to the story (24). Finally, various “atypical” forms of diabetes, mostly with a monogenetic origin, cannot be cured by lifestyle change alone (although lifestyle change has a major impact on metabolic control even in those patients) due to their underlying cause (25). In conclusion, lifestyle change can restore the pathobiology of “typical” T2D to normal in patients who still have sufficient insulin secretory capacity.

### Systems Flexibility As Characteristic of Metabolic Health and Disease

The metabolic state of patients with type 2 diabetes is routinely quantified in a symptomatic manner, i.e., by the fasting plasma glucose concentration (the acute symptom) and HbA1c (the accumulated symptom). Yet, these are the consequences of a complex network of interactions between food intake and fuel metabolism, involving multiple organs and biochemical pathways. For optimal metabolic health, each of these processes needs to function optimally. We argue that effective treatment of type 2 diabetes requires appropriate quantification of the multiple processes leading to disease itself, its progression, and its cure. In this context, it is important to note that type 2 diabetes is not a uniform disease. There are probably many subtypes with their own pathophysiology, born out of specific gene–lifestyle interactions (26). If this is the case, there is no optimal “one size fits all” treatment of type 2 diabetes. Each disease variant most likely requires its own therapeutic approach.

Most of the metabolic processes that go awry in type 2 diabetes have to function in a continuously changing environment (diet, infections, stress, temperature, exercise, etc.) where they strive to maintain internal homeostasis by adapting to these changes, usually in an hour–day timescale. Under chronic external stress (month–year timescale: high calorie diets, chronic inflammation), two major adaptation processes occur. On the one hand, the molecular physiology that regulates the acute stress response reactions accelerates to maintain homeostasis of the important parameters, possibly at the cost of other parameters. During caloric excess, plasma glucose, triglycerides, and inflammatory markers are initially maintained at normal levels, as a result of elevated concentrations of insulin, glucagon, and other regulatory mechanisms (27). However, prolonged caloric excess invokes adaptive processes like storage of excess calories in the form of triglycerides, which eventually may have negative consequences (obesity, ectopic adipose deposits, insulin resistance, inflammation, and eventually impaired insulin secretion). We call this phenotypic or systems flexibility (28). Disease onset occurs when and where one or more of these adaptive processes fail. Importantly, diet and lifestyle play both a negative (caloric excess) and positive role: many nutrients serve specifically to optimize these “flexibility processes” (29). Already in 2002, the Diabetes Prevention Program demonstrated in a large 4-year study that, although metformin and lifestyle both were effective in maintaining fasting plasma glucose, the plasma glucose response to an oral glucose tolerance test (OGTT) (a core aspect of phenotypic flexibility) was more efficiently restored by lifestyle change than medication (13).

**Figure 1:**
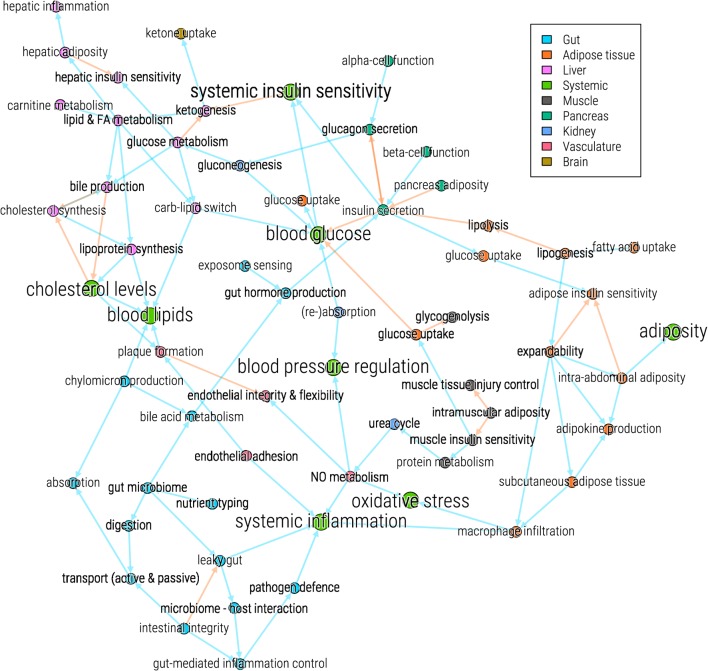
Systems view on processes involved in glucose control and type 2 diabetes. The processes (nodes) are colored according to their involvement with organs (see legend). Blue arrows (edges) indicate a positive effect of one process on another, red arrows indicate a negative effect.

Type 2 diabetes, from a systems flexibility mechanistic perspective, thus can be caused by the (partial) failure of many combinations of processes involved in maintaining homeostasis (29). Pancreatic insulin secretion, insulin sensitivity of liver, muscle, and various adipose depots, intestinal permeability, and lipoprotein metabolism are some of the processes that need to maintain their flexibility. Figure 1 presents an overview of all major processes involved in glucose control and type 2 diabetes. It demonstrates how many processes are connected. A comprehensive overview of these processes is presented in Ref. (30).

## Quantification of Systems Flexibility: Stress Response Biomarkers

Current diagnostic procedures primarily focus on symptoms of T2D. However, parameters such as glycemia, lipidemia, and blood pressure fail to capture the mechanistic underpinnings of the disease. It is imperative to map out the metabolic anomalies that drive these (plasma) markers in detail, if we wish to truly cure T2D (31).

Due to a wide variety of reasons (genetic, epigenetic, exposure, diet, stress, exercise, etc.), individuals differ in their “wiring” of phenotypic flexibility, will react differently to acute and chronic (metabolic) stressors, and develop a personal trajectory of metabolic–inflammatory health and disease. Appropriate therapeutic intervention requires identification of the individual “weak spots” of systems flexibility, allowing personalized advice. The weak spot may be impaired triglyceride storage in adipose tissue resulting in a fatty liver for one (32), for another it may be impaired excretion of VLDL particles from the liver due to a shortage of choline, resulting in a fatty liver (33). A third person may accumulate liver fat due to a shortage of carnitine, causing inadequate fatty acid oxidation (34), etc. Each of these processes needs to be mapped out to carefully design a specific (food and lifestyle based) therapy (35). As an example, individuals with a muscle insulin resistance subtype profit specifically from a Mediterranean diet, while those with predominant resistance of the liver profit from a low-fat diet (36).

To better quantify the specific underpinnings of metabolic derangement, i.e., subtyping of phenotypic flexibility, standardized metabolic stress tests are a promising option (31, 37). Multi-biomarker panels are needed to reflect well-defined and -accepted health-related processes (Table 1) that can be used as diagnostics of systems flexibility by quantification in response to standardized stress testing. This type of method now allows to quantify the relative contribution of many relevant processes in pancreas, liver, muscle, intestine, adipose tissue, vasculature, and kidney in type 2 diabetes, within a single metabolic challenge test (38) based on a multiomics assessment of these changes (39–41). The “amplitude” and the “duration” of disturbance (time needed to get back to homeostatic conditions) are taken as readout. It is of critical importance for proper diagnosis to develop such standardized (metabolic) stress tests.

**Table 1:** Type 2 diabetes subgroup (process) dependent diagnosis–intervention strategies.

T2D subgroups (see Figure 2, based on processes involved)	Diagnosis (i.e., parameters of the biopassport)	Potential interventions
1. Pancreatic β-cell function (impaired insulin secretion)	Oral glucose tolerance test (OGTT) or challenge test: disposition index	Fasting-mimicking diet (FMD); β-cell protective nutrients (MUFA, protein, vit. K, Mg, leucine); β-cell protective drugs (TZDs, GLP-1 analogs, DPP4-inhibition)

2. Muscle insulin resistance (decreased glucose uptake)	OGTT or challenge test: muscle IR index, HbA1C, 2-h glucose	Physical activity (resistance training); Mediterranean diet; low-glycemic index diet; low-carb diet; low refined sugar; fiber (arabinoxylan, alpha-cyclodextrin, resistant starch, beta-glucans)

3. Hepatic insulin resistance (decreased glucose uptake, but increased production and release)	OGTT or challenge test: hepatic IR index, fasting glucose	Low (saturated) fat diet; weight loss; very low-caloric diet; intermittent fasting; wholegrain; choline; carnitine; resveratrol; cinnamon extract; metformin

4. Adipocyte insulin resistance and lipotoxicity	Basal adipocyte insulin resistance index, non-esterified fatty acids, visceral and ectopic fat percentage	Intermittent fasting; FMD; α-lipoic acid; poly-unsaturated fatty acid/SFA balance; omega-3 FAs; TZDs; acipimox

5. Vasculature	Blood pressure, LDL-cholesterol, HDL-cholesterol, fasting, and post-prandial triglycerides	DASH diet; low-sodium diet; wholegrain; fiber (pectin, β-glucan); beet root (extract); lycopene; Vit. C; Vit. K; cocoa flavonols; hydroxytyrosol (olive oil); monacolin K; coenzyme Q10; grape seed extract; chitosan/phytosterols; *L. reuteri* NCIMB 30242; statins; blood pressure lowering medication

6. Chronic low-grade inflammation	CRP, total leukocytes, cytokines	Physical activity; fish oil/n-3 fatty acids; Vit. D; Vit E.; Mg; flavonoids; curcuminoids; salicylates; TNF-α inhibitors

*Currently, six processes involved in T2D are identified, and for each of them a biomarker approach to quantify the process, and an intervention strategy to optimize/restore, is suggested*.

### Toward a “360° Diagnosis”

Apart from the above-mentioned extension of biomedical diagnosis to “systems flexibility subtyping,” other factors or criteria are important for further subtyping or personalization in lifestyle interventions for type 2 diabetes subjects. Examples are personal motivation, diet or lifestyle preferences, contextual (e.g., environmental, social, or economic circumstances), health literacy, psychological stress level of a person’s daily life. Ideally, all of these factors are taken into account in an integrated personalized treatment and lifestyle change.

Such a “360° diagnosis” provides input into the shared decision of patient and professional on how to act and why. For example, it is well established that psychological comorbidities (depression and anxiety disorders), socioeconomic factors (education, debts, shift work), health literacy, and personality traits (deficits in problem solving or coping skills) can all hamper lifestyle change, self-management, and drug adherence (42) (Table 2). More so, it might be wise for some patients to first tackle mental disorders, debts or unemployment, and then actively work on diet or exercise.

**Table 2:** Examples of diagnostic parameters relevant for type 2 diabetes in each of the four areas covered by 360° diagnosis.

Bio	Psycho	Social	Spiritual
Metabolic status	Dietary preferences	Peer pressure	Eudaimonic well-being
Systems flexibility	Stress resilience	Food/sports availability	Life goals
Dietary intake	Personality type	Family habits	Worldview
Physical activity	Coping styles	Work environment	Religion
Genetics		Health literacy	Mindfullness
		Finances	Gratefullness

Furthermore, type 2 diabetes follow-up should not only include biological measures of systems flexibility but also an inventory of critical patient characteristics to set the stage for comprehensive, integrated care (42). The Grid Enabled Measures database has cataloged many of these parameters for obesity and diabetes treatment in the behavioral, biological, environmental, and psychosocial areas^1^ and, depending on goal, type of intervention, selections can be applied.

Future biomarker developments in T2D may include the combination of personal genome, phenotype, and environment (“Exposome-Phenome-Diseasome Associations”) as proposed by Vasan and Benjamin (43), based on the concept of network interactions (“diseasome”) of all relevant information, spanning from genetics to social interactions, already proposed 10 years ago (44).

## The Interventions

### Only Systems Interventions Will Work

As explained above, type 2 diabetes is multifactorial and thus requires systems interventions. This implies that all underlying causes are quantified and addressed in a personalized and (chrono-)logical order. These causes fall into categories spanning the range of biological, psychological, sociological/environmental, and spiritual domains. Interventions likely fail if one or more of these domains are not properly addressed, or when the interaction between each of these domains is not well understood. It also means to go beyond the treatment of symptoms, but rather to understand the syndemics behind health problems (45). Each type of intervention is addressed in detail below. Some common features that should mark all interventions include

1. The efficacy of a systems approach is based on its individual components and a tailored analysis of the best combination of components (15).
2. Interventions for lifestyle-related diseases need to be based on “self-empowerment.” Most externally imposed interventions are not sustainable. This is the case for the large majority of interventions, with the LookAHEAD study as prime example (46).
3. Interventions always aim to improve flexibility and/or resilience. This holds for both the biology/physiology and psychosocial aspects.

### Dietary Interventions

#### (Chronic) Calorie Restriction

There is no doubt that caloric restriction and weight loss ameliorate metabolic anomalies in patients with T2D (47, 48). Indeed, loss of 5% of bodyweight or more reduces HbA1c, lipoprotein levels, and blood pressure. Restricting energy intake to 600 kcal/day for 8 weeks normalizes beta-cell function and hepatic insulin sensitivity in obese type 2 diabetics, coinciding with reduction of hepatic and pancreatic fat content (23, 49, 50). These data clearly indicate that type 2 diabetes is a reversible disease, which can be cured by appropriate dietary measures (although the genetic predisposition obviously never disappears). Interestingly, upon publication of the data, many T2D patients reported similar effects of very low calorie intake in their daily life practice, demonstrating that the disease can also be reversed in a non-research self-empowerment setting (51). (Severe) caloric restriction is difficult to sustain, if not plain deleterious in the long run. Thus, the “DiRECT” study was designed as a 4-year demonstrator of the above-mentioned caloric restriction, where the treatment of 149 type 2 diabetes patients consisted of withdrawal of antidiabetic and antihypertensive drugs, total diet replacement (~850 kcal/day formula diet for 3–5 months), stepped food reintroduction (2–8 weeks), and structured support for long-term weight loss maintenance. The first-year results are published and show remission of type 2 diabetes (i.e., HbA1c below 6.5% without medication) in 48% of the subjects, while the control group (standard care) showed 4% remission. Interestingly, the remission was associated with weight los, with subjects losing more than 15 kg showing 86% remission (52).

#### (Intermittent) Fasting

Fasting, including caloric energy restriction and different intermittent fasting regimes, has been shown effective in weight loss, improving insulin sensitivity, and decreasing cardiovascular risk in both non-diabetic and diabetic subjects (53–56). A study by Halberg et al. in which participants followed an alternate day fasting scheme while maintaining body weight and fat mass, still demonstrated increased insulin-mediated whole body glucose uptake rates, insulin-induced inhibition of adipose tissue lipolysis, and increased plasma adiponectin levels (57). This suggest that the positive effects of intermittent fasting are not solely attributable to weight loss, but are also driven by other mechanisms enhancing metabolic/phenotypic flexibility. The profound metabolic benefits of *intermittent and periodic* fasting have been well documented in preclinical experiments. For example, alternate day fasting (i.e., complete fasting for 24 h alternated with 24 h periods of *ad libitum* intake) fully reverses the high insulin and glucose levels of db/db diabetic mice to normal, despite similar overall food intake and stable bodyweight compared to *ad libitum* fed animals (58). However, fasting every other day is probably even less realistic than chronic calorie restriction and its effects in humans are poorly understood (59). The periodic and prolonged use of the so-called fasting-mimicking diets (FMDs) may offer an effective and safe alternative for the treatment of T2D in humans. FMDs are meal replacement plans, mimicking the endocrine and metabolic effects of fasting while containing modest numbers of calories. The characteristics of these diets that are critical for appropriate copying of the effects of total fasting (despite considerable calorie content) are lack of refined carbohydrate, a very low-protein content and high levels of healthy fats, all from plant-based sources (60). Both animal and human studies suggest that FMDs can be applied as infrequently as once a month for 5 days, requiring approximately 50% reduction in calories to be effective in promoting strong effects on metabolic syndrome risk factors (61, 62). Remarkably, and in keeping with previous reports (58), animal studies indicate that the effects of the periodic FMD on disease risk factors does not require overall calorie restriction, since mice on the FMD consumed the same number of calories per month as mice on the *ad libitum* diet (61). The molecular mechanisms that underpin the benefits involve the persistent endocrine and metabolic shifts typically induced by fasting: (1) reduction of (bioavailable) insulin growth factor-1, insulin, ectopic fat storage, and endogenous glucose production; (2) increased adipose lipolysis and fat oxidation; and (3) use of glycerol and ketone bodies instead of glucose as preferred carbon sources (59). Notably, recent experimental evidence suggests that periodic use of FMDs can drive beta-cell regeneration to restore insulin production in animal models of type 2 (and type 1) diabetes (24). The potential benefits of intermittent fasting could lie in “flexibility training”; i.e., the frequent switching between metabolic modes, from glucose to free fatty acids and ketone bodies as energy source, i.e., inherent to fasting (59). This finding is in line with the positive impact of intermittent fasting on insulin sensitivity, inflammatory markers, oxidative damage, and stimulation of autophagy (59, 63, 64).

Some studies, however, did show adverse effects of intermittent fasting in healthy, non-obese subjects, including increased levels of free fatty acids and impaired glucose tolerance (65, 66), suggesting that intermittent fasting should only be applied in metabolically inflexible persons.

No studies as yet have been performed that compare effectiveness of an isocaloric intermittent fasting scheme and caloric energy restriction, or healthy diet intervention. Such studies are required to confirm the ability to “train” metabolic flexibility with intermittent fasting.

Collectively, currently available evidence strongly supports the clinical potential of periodic fasting as an effective and safe alternative to chronic restriction of calories. FMDs may be a feasible mode to implement this therapeutic strategy.

#### Macronutrient Composition

The macronutrient composition of food is relevant for metabolic control as well. As mentioned above, in theory, abolishing the need to store excess glucose by low-carbohydrate (particularly sugar and starch restricted) diets obviates the need for glucose lowering medication, simply because there is no glucose to be stored. Numerous studies have been performed with low or poorly digestible carbohydrate in type 2 diabetics [e.g., Ref. (67–71)]. Follow-up was 1 year in most of them. The data suggest that, in the short term (4 weeks to 6 months), carbohydrate restriction is effective in terms of glucose and weight control. However, in the long run, the effects in terms of bodyweight, glucose control, and lipid profiles of low-carbohydrate diets do not appear to be superior to those of high-carbohydrate diets (72). A meta-analysis of intervention studies (in diabetic and non-diabetic humans) revealed that substitution of carbohydrates with poly-unsaturated fatty acids significantly reduces plasma HbA1c and insulin, while replacing carbohydrate with saturated fat reduces only insulin levels somewhat (73). Besides carbs and fats, effects of dietary protein on diabetes type 2 also received a lot of attention in literature. The acute insulinotropic effects of especially whey protein have been quite well established (74). This effect seems to be mainly driven by an increase in GLP-1, as well as decreased gastric emptying (75, 76). However, increased insulin production is not a beneficial effect if not accompanied by increased insulin sensitivity. Overstimulation of the pancreas and stimulation of gluconeogenesis by high-protein diets may in the long term even result in increased (muscle) insulin resistance (77), if not compensated by increases in lean muscle mass or weight loss (78, 79). It can thus be concluded that manipulating the macronutrient content of the diet without restriction of calories does not cure T2D in the vast majority of patients, although it can improve the diabetic phenotype to a certain extent.

#### Micronutrients and Non-Nutrients

Many micronutrients and non-nutrients were shown to improve glucose control, interacting with a large variety of pathways and processes. Interestingly, almost all processes involved in maintaining systems flexibility (see above) are targets of micro- or non-nutrients. These are summarized in many dedicated reviews (17, 29, 80, 81). Micronutrients are involved in ectopic lipid disposition [e.g., choline deficiency reduces fatty acid oxidation (82, 83) and hampers VLDL particle synthesis, and thus stimulates fatty liver (84)]. Various nutritional therapies were shown to be efficacious for non-alcoholic fatty liver disease (84). Nutritional anti-inflammatory compounds (e.g., polyphenols, omega-3 fatty acids) contribute to reversing type 2 diabetes, although primarily in combination with other interventions (85), and in a gender-specific manner (86). Insulin secretion is optimized by zinc (87), magnesium (88), and vitamin D (coinciding with its effect on inflammation and glucose control) (89). In theory, a healthy diet should suffice in the needs of these micronutrients. Given the fact that type 2 diabetics usually do not have a track record in healthy eating, targeted prescription of micronutrients and non-nutrients may be required. Diagnosis of the malfunctioning components of systems flexibility in T2D (or any other disease) will allow the design of specific (nutritional) therapies.

#### Personalization

One of the reasons for the lack of consistent, significant effects of the dietary interventions described above probably pertains to the fact that people are not born or raised equal. Moreover, as described in the previous paragraphs, systems diseases require systems diagnosis based on quantifying phenotypic flexibility. This reveals the underlying physiological disease cause(s), embedded in a complex network of metabolic and inflammatory processes. For optimal systems flexibility, each process needs to function optimally. In type 2 diabetes, many organs and processes can contribute to disruption of (glucose) metabolism (90). The degree of insulin sensitivity of the three main organs involved in maintaining glucose homeostasis (muscle, liver, and adipose tissues), and the degree of insulin excretion by the pancreas can be assessed by measuring glucose and insulin at 30-min intervals during an OGTT, together with fasting free fatty acids (16). The severity of insulin resistance can differ between the various organs, and different interventions may have organ-specific effects related to increasing insulin sensitivity (91), as demonstrated by the example of treatment of type 2 diabetic patients with a very low-caloric diet (VLCD) or physical exercise. When insulin resistance primarily affects muscle, physical exercise rapidly restores glucose tolerance (92). In contrast, patients with insulin resistance of the liver respond particularly well to VLCD (23). However, when β-cell capacity is not sufficient, neither VLCD nor physical exercise will fully reverse glucose tolerance (23, 93–95). Similar tissue-specific metabolic effects of distinct interventions were also demonstrated in the CordioPrev cohort, where the Mediterranean diet specifically improved glucose metabolism in T2D patients who predominantly had muscle insulin resistance, while a low-fat diet specifically benefited patients with liver insulin resistance (36). These observations are supported by mechanistic data on metabolic flexibility, demonstrating differential molecular routes for insulin resistance in distinct organs (96). This begs for systems interventions based on diagnosis of decreased flexibility of (tissue-)specific health-related processes. In taking this concept further, toward all aspects of phenotypic flexibility, Table 1 gives an example of how we envision systems diagnosis and related interventions for T2D. Many systems biology based examples of nutrient–flexibility–health relationships and the ways in which they can be personalized have been reviewed (29). In the area of hypertension, an “integrative approach” was proposed, using a multitude of food products and supplements on top of the DASH diet (97). Recently, an international panel recommendations for the prevention and management of metabolic syndrome with lifestyle also showed clear scientific evidence for lifestyle treatments based on weight loss and increased energy expenditure through physical activity, for a Mediterranean-type diet, with or without energy restriction, as well as for other similar dietary patterns, next to quitting smoking, reducing intake of sugar-sweetened beverages and meat products (17).

### Physical Activity Interventions

There is strong evidence for the beneficial effects of physical activity on insulin resistance, type 2 diabetes, dyslipidemia, hypertension, and obesity (98, 99). Even a single exercise bout improves blood pressure, glycemia, carbohydrate oxidation during exercise, and fat oxidation after exercise (100). Physical activity programs were shown to be effective in NAFLD treatment (101). Specific physical activity programs achieve specific metabolic health improvements. For example, high-intensity intermittent exercise was shown to specifically reduce liver fat (102). Sixty minutes of walking resulted in a 75% increase of whole body insulin sensitivity (103). On the other hand, just like overeating, lack of physical exercise as such is associated with a number of lifestyle-related diseases (104–106). Physical activity programs can be targeted to specifically reduce ectopic fat and thus become part of a lifestyle program for T2D. A meta-analysis indicated that especially endurance training (aerobic exercise) had an effect on visceral fat and possibly intrahepatic fat in type 2 diabetics (107).

Mechanisms involved are restoration of metabolic flexibility, the oxidative part of insulin resistance, and reducing the reactive oxygen species production. Also, physical activity attenuates inflammation in a positive manner, which contributes to management and reversal of type 2 diabetes (108). Most of these mechanisms involve the mitochondria and are especially valid for the muscle (109), suggesting a specific benefit of physical activity for patients with muscle insulin resistance.

### The Role of Medication in Lifestyle Therapy

The current pharmaceutical approach to diabetes care is to assist the patient in maintaining glucose, lipid, and blood pressure control. Metformin primarily decreases hepatic glucose production. Thiazolidinediones assist in fatty acid storage in adipose tissue, thereby increasing the use of glucose as energy source and reducing ectopic fat storage. Sulfonylureas stimulate insulin secretion, DPP4 inhibitors prolong the half-life of insulin-stimulating hormones, and exogenous insulin facilitates organ glucose uptake. Medication is prescribed depending on the stage and severity of the disease. Yet, none of these medications addresses the root cause of T2D and will thus not cure the patient.

Plant-based diets almost immediately reduce the need for insulin in “insulin-dependent” type 2 diabetics (110), because absorption of complex carbohydrates from plants just modestly increases plasma glucose levels, which obviates the need for insulin to facilitate glucose storage. Very strict low calorie (600 kcal/day) dieting restores plasma glucose concentrations to normal in T2D patient in 1 week (23). Yet, although such intervention initiate the reversal of type 2 diabetes, the trajectory toward real cure (i.e., restoration of organ insulin sensitivity and beta-cell insulin excretion) will take much more effort and time. Meanwhile, medication may be needed. Therefore, a lifestyle intervention as therapy for T2D will need to be supervised by medical professionals. Notably, patients who choose to adopt lifestyle changes to treat their T2D often need to convince their doctors to adapt their medication. Doctors generally consider medication as required for glucose management (111).

In a fully integrated approach to cure of T2D, lifestyle and medication should (sometimes) be combined. Pharmaceutical strategies should be designed to support optimal reversal of tissue insulin resistance and beta-cell failure, i.e., a combined “precision medicine/precision lifestyle strategy.” As described above, type 2 diabetes essentially needs a systems view, systems diagnosis, and systems therapy to restore insulin sensitivity of all relevant tissues (liver, muscle, and adipose), and reactivation of beta-cell insulin secretory capacity. The extent to which insulin sensitivity and/or action are compromised in each of these tissues may differ between patients. Therefore, the treatment strategy should be tailored to personal disease characteristics. For example, hepatic insulin resistance due to hepatosteatosis in a relatively lean subject may be due to impaired fatty acid uptake by subcutaneous fat or by excessive consumption of refined carbohydrates. Combining a PPAR agonists with restriction of sugar (and alcohol) intake could be a beneficial “food-pharma” couple.

Depending on the disease progression, two other aspects related to medication need to be taken into account. First, not all aspects of systems flexibility may be fully restored, as irreversible damage may have occurred. For example, impaired insulin production can be reversed by reducing pancreatic fat storage and glucose toxicity (112), but beta-cell damage may not be fully reversible. Second, not all comorbidities and pathologies resulting from hyperglycemia are reversible, although the causal drivers (obesity, hypercholesterolemia, hyperglycemia) can be reversed. Indeed, cardiovascular, renal, ocular, and neural complications might need specific medication.

### Sustained Behavior Change

A lasting change in health requires sustainable lifestyle changes of individuals and active self-management. Behavioral change interventions are designed to affect the actions that individuals take with regard to their health. These interventions can be directed at the individual *via* psychological determinants of behavior, or target their social or physical environment to create a supportive environment.

First of all, for sustained behavior change, various phases need to be confronted. Initiation of behavior change is by no means a guarantee for continuation. Rothman et al. suggested four phases of behavior change: behavioral initiation, continuation, maintenance, and habit (Table 3) (113). Before the phase of initiation of new behaviors, old habits (like unhealthy eating) are prominent. In the phases of initiation and continuation, the newly adopted behaviors can be in conflict with old patterns, and within this phase the relapse to old habits is likely. If the new behavior becomes the dominant response across context, and people are able to override previous automatic responses, maintenance, and habit become likely. Each phase needs tailored behavior change strategies (113). Whereas the initiation phase is mainly based on outcome expectations and efficacy beliefs, continuation depends on the reward gained from the behavior and self-regulatory skills like planning, sustained self-efficacy, self-regulatory effort, including self-monitoring (114, 115). Maintenance has been linked to factors like self-identity, satisfaction/enjoyment of the new behavior (116). A new habit is achieved when the newly formed behavior has become the automated response.

**Table 3:** The various phases in behavioral change, together with the individual’s perspective and coaching methods.

Attaining habits	Individual	Coaching methods
Onboarding	Identification—who am I	Motivational interviewing and tailoring

Initial effort	I want this I can do this I believe the new behavior will help me to realize my goals I need to get rid off my old habits	Goal setting Action planning Modeling De-conditioning

Continue effort	I know my obstacles and I can overcome them I can maintain the new behavior	Problem identification and solving Coping planning and self-monitoring, self-evaluation

Sustained effort	I enjoy the new behavior and its results I can do this everywhere, anywhere, anytime	Self-monitoring, self-evaluation Prompt repetition in various contexts

Habit	It is part of who I am	☺

Behavior change interventions have been effective in creating changes in physical activity and dietary intake (117, 118) and have resulted in changes in HbA1c levels (119), especially for those with a higher baseline HbA1c and for interventions of at least 1 year. Behavioral maintenance of physical activity and dietary behaviors can be achieved when these interventions are conducted over a longer period (>24 weeks), include face-to-face contact, multiple intervention strategies, and follow-up prompts (120). The effectiveness of these interventions depends on the balanced combination of behavior change strategies to promote and support behavior change (118).

Studies that have effectively supported type 2 diabetes patients have been based on:

– prompt focus on past success (identifying and emphasizing successful behavior change from the individual’s past);
– barrier identification/problem-solving (identifying salient barriers to physical activity for the individual and strategies to overcome them);
– use of follow-up prompts (such as reminder postcards or motivational telephone calls);
– provide information on where and when to perform the behavior (individuals are given explicit information on locations, times, and opportunities available locally for changing physical activity behavior);
– prompt review of behavioral goals followed by revisions or adjustments (121);
– maintenance motives (focusing on reward of changing the behavior) (122);
– environmental restructuring (to facilitate the desired behavior) (123).

Lifestyle change is not an effortless or innately pleasurable process for most people. Thus, engaging in healthy behaviors needs to be fulfilling or rewarding—until the long-term benefits of the new healthy lifestyle become manifest and the new lifestyle has become habitual. Reward-based reinforcement is a much-researched learning theory strategy to promote behavior change, which includes vicarious reinforcement (imitating behaviors of others who have experienced rewards due to the behavior) and incentives [which can be (financial) presents]. Incentive-based interventions have been successfully applied to various areas of behavior change, ranging from smoking cessation and physical exercise to counseling attendance and medication adherence (124). Reward-based approaches address the principle that people are prone to succumb to instant gratification, rather than investing for the future. Some people are more prone to instant gratification, and it is often associated with the immediate reward of eating. Meta-analytical evidence shows that providing (financial) incentives is an effective strategy in health behavior change (124). As a consequence, reward-based programs have gained a lot of interest among policymakers and governments. Both the United States and the United Kingdom governments advocate the use of incentives to encourage healthy lifestyle choices and have started large-scale reward programs that target vulnerable populations. In South Africa, health insurer discovery has developed an advanced reward program that collaborates with businesses to form a “lifestyle loyalty” program. Participants earn reward points for all kinds of health activities and behaviors, and the points can be exchanged for goods and discounts at local and national businesses. Active participation in the program showed to increase exercise, healthy food purchases, and decrease healthcare costs. A comparable approach is adopted in the Netherlands for cardiac patients and could be easily adjusted to type 2 diabetes.

Persuasiveness is a key element to promote behavioral maintenance and adherence to interventions (125). Although monetary rewards may function as incentives, other types of persuasive design methods of [ehealth (eHealth)] interventions have been examined as a means to increase engagement with interventions and behavior change. Whereas the behavior-change strategies are mainly targeting primary task support, supporting behavior change, other persuasive design features focus on dialog support, increasing attractiveness of interventions or the reward of behavior change. A particular persuasive strategy is gaming or gamification principles. These so-called serious games are not only meant to entertain but also aim to educate or promote behavior change. Serious games can promote the use and adherence to behavior change interventions. Indeed, a recent meta-analyses examining the role of video games in the promotion of lifestyle changes, suggested that serous gaming is effective, albeit to a limited extend (126, 127). A particular type of gaming has been exergaming or active video games, which are games that require physical activity. These include games, such as dancing games, which generally are done indoor. Exergames seem to be able to contribute to physical activity and bodyweight control, although the effects appear to be limited in uncontrolled settings, and keeping people involved seems a challenge (128, 129). Nevertheless, with the emergence of new types of mobile exergames, for instance, using combined virtual reality and stimulating physical activity at any time and place, exergaming may have potential (130).

Therapeutic alliance is another type of engagement strategy. Therapeutic alliance has been defined as a non-specific feature of treatment, reflecting the extent of collaboration, purposeful action, and emotional connection between a client and therapist. Within the context of interventions guided by healthcare workers, it has been shown that therapeutic alliance increases adherence and effectiveness (131). Also in eHealth interventions, therapeutic alliance has been shown to enhance engagement (132, 133). This certainly is important, as early dropout tends to occur during eHealth interventions.

**Figure 2:**
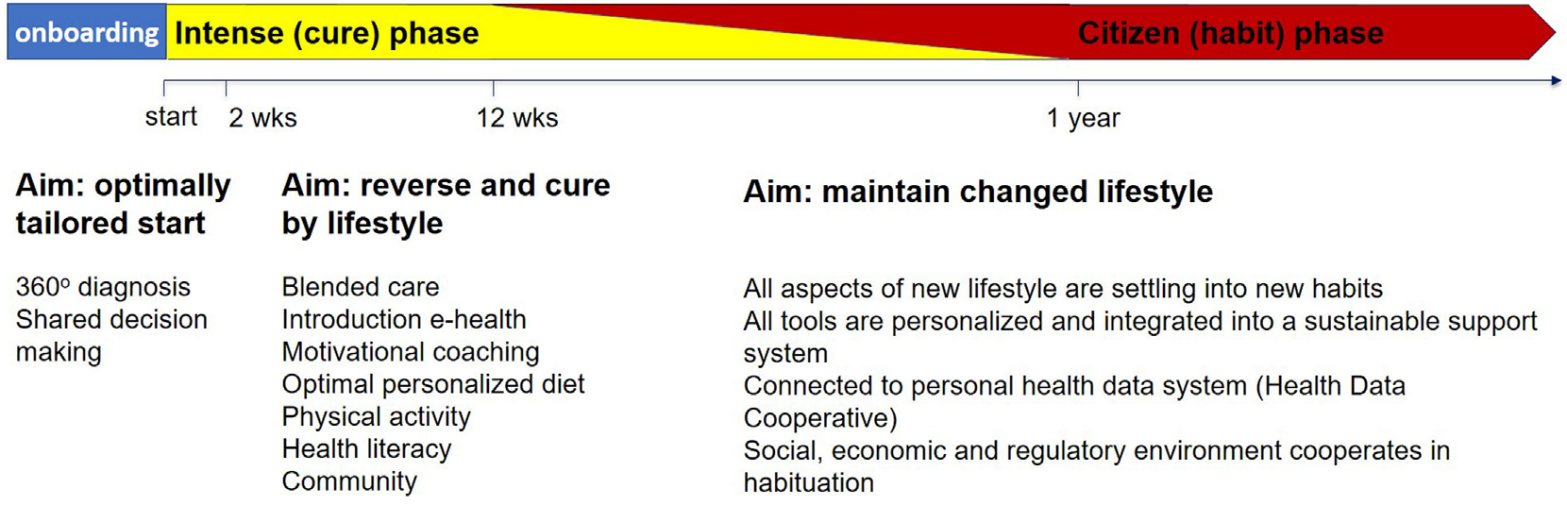
Schematic representation of a personal cure and maintenance trajectory. Depending on the personal 360° diagnosis, the duration and intensity of the cure and maintenance phases and the relative contribution of the components may vary.

Lifestyle-based therapy aims to results at reversing and possibly curing type 2 diabetes. Reversal may be achieved within a relatively short period (weeks to months, depending on intensity of the program and severity of the diabetic state). After this “cure phase,” continued support may be helpful lifelong, both focusing on maintaining an optimal lifestyle and on supporting behavior. Figure 2 presents a schematic overview on the various phases, mentioning some of the important components that can be applied during the various phases.

### Health Literacy

Health literacy in type 2 diabetes is often but not unambiguously associated with glycemic control and other disease endpoints (134). Since care and cure of type 2 diabetes heavily depend on self-management, and since this is a complex matter involving many aspects of information, awareness, reasoning, and knowledge, health literacy is of high importance. As mentioned above, health literacy should be taken into account when diagnosing a patient.

Health literacy methods are the topic of research and evaluations on their efficacy are in progress. Taken together, multiple aspects and approaches are available and real-life implementation in a tailored, if not personalized manner involving all stakeholders needs to be part of the diabetes care and cure agenda (135).

### Structural Interventions for Risk Groups

Besides individual support, structural interventions should be initiated for particular groups of people with T2D that hamper changing their lifestyle due to social–contextual constraints, including neighborhood characteristics, availability of foods, poverty, the local role of primary care, etc. (136). Structural interventions, i.e., interventions aimed to alter the context in which people are living, are likely to promote sustainability and may have additional positive effects on people, besides lifestyle changes and changes in physical health, such as lower experienced loneliness, improved social cohesion, etc. (137). This in particular may be needed for people at risk, e.g., citizens of low socioeconomic gradient, as they are often experiencing a multitude of problems, which need to be targeted by interventions to enable them to effectively change their lifestyles (138). Solutions are sought in community-oriented multidisciplinary primary care interventions in many countries.

### eHealth and mHealth in Type 2 Diabetes

Information and communication technologies, i.e., eHealth, can facilitate health care (139). Particularly in the area of eHealth self-management for chronic somatic conditions, guided and embedded interventions have been shown to be as effective as face-to-face treatment but are usually more cost-effective (140). In type 2 diabetes, innovations in eHealth have a demonstrated potential for supporting patients with self-management behaviors, in particular dietary and physical activity behaviors, and may result in better diabetes outcomes such as HbA1c (141). Innovations that can have positive impacts on self-management behaviors include text messages, smartphone apps, and web-based programs (141). Several systematic reviews showed that telemonitoring can improve HbA1c levels (142–144). Video games, virtual and augmented reality, and wearables are also promising, but individuals should be adequately trained in the use of these technologies (141). Moreover, guided eHealth interventions are usually more effective than non-guided interventions (140), which urges for “blended care” solutions, so that eHealth is part of a structured care plan.

Thousands of mobile (mHealth) apps for diabetes are available for download in App stores (145). Only a small number of the available apps are evidence based (146). Some systematic reviews and meta-analyses showed that mobile phone interventions lead to improvements in glycemic control and self-management (147, 148). Apps for diabetes self-management typically share a limited number of basic functions, which can be classified into several categories, e.g., self-monitoring, education, support, alerts and reminders, and communication (149). To evaluate the effectiveness of the different functions on glycemic efficacy, Wu et al. developed a taxonomy of apps for diabetes self-management (150). In line with earlier studies, they showed that the use of mobile app-based interventions yields a clinically significant HbA1c reduction among adults with type 2 diabetes. Having a complication prevention module and/or a structured display was associated with a greater HbA1c reduction. Other functions (medication management, generation education, personalized feedback, communication, potential-risk intervention with clinical decision-making) were not associated with greater HbA1c reductions.

eHealth and mHealth can be applied both in the management and prevention of diabetes and thus should be embedded in the continuum of prevention, care, and cure. The American Diabetes Association stated that eHealth technologies, such as Internet-based social networks, distance learning, DVD-based content, and mobile applications may be a useful element of effective lifestyle modification to prevent diabetes (151). The US Center for Disease Control and prevention and the Diabetes Prevention Recognition Program have begun to certify eHealth- and mHealth-based modalities as effective media for diabetes prevention interventions that may be considered together with more traditional face-to-face and coach-drive programs (blended care). Apps for weight loss and diabetes prevention have been validated for their ability to reduce HbA1c in the setting of prediabetes (152). Traditionally, most weight loss apps focus on reducing caloric intake, but for people with (pre)diabetes it is more important to make food choices that induce normal post-meal glycemic responses (153).

mHealth can be greatly improved when ICT technologies are combined with evidence-based behavior change interventions. It has been shown that eHealth interventions that use more behavior change strategies were more likely to effectively change health behavior (physical activity, healthy eating, weight loss) (118, 154). And through, e.g., cultural tailoring (e.g., tailoring to gender, age, religion, ethnic background, or health literacy) acceptance, loyalty, and effectiveness of digital health can be improved (155). Furthermore, computer-tailored interventions have become increasingly common for facilitating improvement in health behaviors. Dynamic tailoring (where the intervention variables are assessed before giving feedback) was shown to be more efficacious than static tailoring (all feedback is based on one baseline assessment) and has long-term effects (156). For all eHealth interventions, guided treatments are usually more effective than non-guided treatments (140).

Combining face-to-face counseling with extended care (*via* dynamically tailored support) has a potential to increase the effectiveness of T2D management (157). There is a clear need for delivery systems to use team-based models and engage patients in shared decision-making (SDM), where patients and providers together make healthcare decisions that are tailored to the specific characteristics and values of the patient. It has been demonstrated that such an approach leads to patients reporting better understanding of diabetes and showing improved hemoglobin HbA1c values, while healthcare providers reported the SDM aids increased cohesion among team members (including patients) and facilitated patient education and behavioral goal setting (158). In a large pragmatic trial making use of motivational interviewing, SDM, and collaborative goal setting in chronic conditions, a striking difference in mortality rates was found after 2 years of telephone-based health coaching (OR = 0.64; *p* = 0.005), which was achieved with an average of 12.9 calls per patient (159).

### The Value of a Timeline of the Health and Behavior Trajectory

Ideally, biomarkers and diagnostics develop into two dimensions. First, from a single process to the complete quantification of health, including flexibility (“systems flexibility biomarker,” described above), and second, along the timeline of an individual’s health trajectory, building the life story of systems flexibility, a personal “biopassport.” Loss of systems flexibility is a process that develops over the timespan of many years. Interventions are most successful in early stages, when full reversal and cure is possible. The storage and availability of biomarker data has been common practice in longitudinal cohorts, but the translations of its results into health care is a tediously slow process. Also, personal health(care) data are usually not available in a structured and understandable manner for the citizen/patient to valorize for his personal health. Since lifestyle-related health is primarily dependent on self-management and self-empowerment, it is vital that the citizen/patient has access to all relevant health data and information (8). If biomarkers of phenotypic flexibility are the key in optimizing metabolic health and in prevention and treatment of metabolic diseases, they need to be measured in regular intervals. At this moment, this is not practical and not affordable, and moreover most healthcare systems neither focus on nor reimburse preventive diagnostics. Therefore, new diagnostic applications need to be developed which are cost-effective, minimally invasive in preferably “do-it-yourself” applications. Developments, both in ICT (personal health portals) and in diagnostics (“gadgets,” dried blood spot diagnostics, etc.) are rapidly disclosing this area. Various sensors (e.g., wearable, on-phone, at home) and other measurement devices (e.g., glucose monitors, weighing scale) have become cheaper and more user friendly. The challenge is to use these techniques in a complementary manner (160). As an example, the “Nutrition Researcher Cohort” (161) started this movement in 2011. However, this cohort encountered major obstacles related to research ethics as it appeared to be virtually impossible to merge personal health data collection with “citizen-science” research, and a further development of “participant led research is needed” (162). The NIH “All of Us” cohort as part of the Precision Medicine Initiative is professionalizing this movement (163), although not focusing on lifestyle. New, partly commercial activities are maturing this area (11). Also, “big data” and artificial intelligence-derived solutions are emerging for clinical decision support, like IBM’s health analytics (164). Finally, apart from the above-mentioned developments that shift diagnosis away from the traditional medical domain toward self-empowerment, other more unexpected developments arise. Internet search engine data analysis is rapidly becoming a powerful tool for surveillance (165). These opportunities now move from surveillance to research tools (166), but it may take some time before they become applicable for personal diagnosis due to privacy and ethical constraints. A biopassport is the ideal starting point for the design of lifestyle-based personal health optimization and self-empowerment strategies. The biopassport can be extended with the above described “360° diagnosis.” eHealth applications can be tapped into the biopassport to deliver advice and guidance. Ecological Momentary Assessment/Intervention application can be embedded in and will enrich the biopassport (167). Essentially, the personal availability of the timeline of health data will trigger a wealth of applications and economic developments that support personal healthy living and lifestyle-based therapies.

### Health Data Cooperatives As Ultimate Platform of Health Democracy

Science, business, and society now begin to realize the value of citizen-owned health data (8). Numerous participant or patient centric initiatives have emerged, either from within or outside the healthcare institutions, both commercial and non-profit, and all of them based on Internet and social networking (168, 169). An enormous power is being unlocked in extending and integrating these data, and this is acknowledged by investments in this area through companies like 23andMe and PatientsLikeMe, and activities of most major “big data companies” (170). Of course, the best entity to valorize citizen-owned health data for any purpose (science, health care, or economy) should be the legal owner, i.e., the citizen itself. The “Health data Cooperative” (171) may be an attractive model for this purpose, as it is the most democratic shared ownership and decision-making legal entity available. This would unlock the value of personal health data for the benefit of personal and public health, facilitate an optimal merge between healthcare quality management and research, and provide the citizen/consumer/patient with the power to become the center of health care. If indeed the economic value of health data can be invested for the genuine benefit of the citizen/consumer/patient instead of commercial stakeholders, this has the potential to disrupt the current healthcare system into a citizen-centered and self-empowering healthcare system and economy, where services can be implemented that can optimize health, prevent lifestyle-related disease, and cure these using the right tools. Among the right tools should be support of a structured population health management strategy aiming at stratification of the population with T2D according to their risk of various relevant adverse health outcomes. This can be realized with linkage and advanced analysis of (when needed coded and anonymized) routine healthcare data from all domains, resulting in a structured approach of individuals who are member of subpopulations sharing defined risks.

### Toward Integrated “Companion Systems”

Once the above expose materializes in actionable interventions, the type 2 diabetes patients will be overwhelmed by a wide range of advice and information. Generally speaking, these patients have not shown excellence in adherence to advice nor have they all the skills required to adequately deal with a wide range of advice and information. Thus, many components line up for a new failure in implementation and dropout of patients after initial start. Especially, if we look at mHealth we see the number of mobile phone apps for type 2 diabetes is enormous, being the largest application area in the 100,000+ health Apps available in 2014 (145). It is unlikely that any of these apps covers all areas needed, and it is unlikely that any significant percentage of type 2 diabetics will be able to sensibly maneuver among this overwhelming offer.

**Figure 3:**
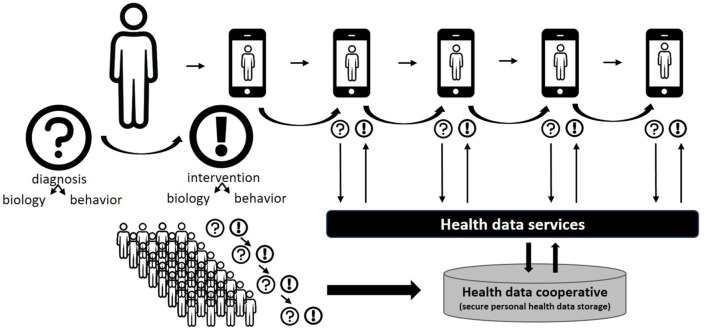
Schematic overview of a “life companion” approach. The citizen–patient interacts with a single ehealth (eHealth) platform (any combination of phone, desktop, life coach, healthcare provider) and receives interventions in all relevant areas (diet and lifestyle, behavior, information, etc.) at the right time in the right message format, based on both initial and continued diagnosis. The intervention is generated by “health services,” i.e., models that exploit personal health and behavior data. Timelines of diagnostic and intervention information are owned by the citizen/patient and may be shared within a community (health data cooperative), thus further strengthening the personal health data service with a “big data” component.

We therefore propose a different approach, where a multitude of overlapping personal advice systems applications are replaced by an integrated ecosystem of health services, provided to the end-user in an “on demand” manner based on real-time needs. Health services can span advice on diet, medication compliance, physical activity, behavioral guidance, health and product information, community building, etc. These services should only be presented to the (ex-) type 2 diabetes patient when relevant and needed, in a format and language that is fine-tuned to the user’s socioeconomic–cultural needs, and optimally facilitate liaison with healthcare professionals. Obviously, these services can become very complex and may include artificial intelligence, ecological momentary assessment and interventions, just in time adaptive interventions and similar approaches. Also, a wealth of personal health data can be used as input, spanning from personal health monitoring to medical records. Yet, none of this complexity should be visible to the end-user, but the interaction with this “life companion” should be minimized to non-intrusive essentials matching the health literacy of the user. Various presentation modes (mobile, desktop, life coaching, or SDM) are possible. Figure 3 presents a schematic overview of the functionalities of a life companion approach. Examples are emerging which combine the layers described above, integrating all aspects of P4-medicine (personalized, predictive, preventive, and participatory) with data- and knowledge drive advice systems in the area of type 2 diabetes (172).

## The Environmental Support

Most of the arguments presented above deal with optimizing the biological interventions within a self-empowerment behavioral change setting. Yet, if we agree that a systems solution is the only way forward toward a cure, the other side of the equation also needs to be addressed, i.e., coping with the diabetogenic environment, and ideally changing the diabetogenic environment (Table 4). Behavioral change technologies connected to eHealth solutions and personal health data valorization are essential in coping with both the biological and environmental pressure to consume calories. Other components of the system entail the environmental support. This includes family, community, and other social structures, primary healthcare centers, other health services, food providers, policies, and regulations. The efficacy of these “population based lifestyle interventions” has been reviewed (173) and evaluated (174), where, six categories of interventions were taken into account, ranging from educational campaigns to taxation. Apart from the efficacy assessment of each of these approaches, a clear statement was made about the need of a combined, “systems” approach (15). The Chicago based South Side Diabetes Program is a rare example of a systems approach taking into account all these levels in a real-life setting (175, 176). This program demonstrates how a multitude of tools (or “interventions”) can be implemented in, and fine-tuned toward a socio–ethnic–economic setting, involving various levels of society. It also demonstrates the complexity and the hurdles of implementing such neighborhood programs outside and independent of research funding. Essentially, for such programs to become effective and sustainable, they need to be fully incorporated in a profitable economic system.

**Table 4:** Type 2 diabetes is a systems disease where solutions need to be found in all three systems mentioned to achieve a transition from care to cure.

Social system	Physiological system	Healthcare system
**T2 diabetes is a “systems disease”**		

Obesogenic environment	Multiple interacting physiological processes	Conflicting stakeholder interests
Limited engagement with health status		No focus on prevention
Social interactions are important for outcome	T2 diabetes initiates when one or more biological processes lose flexibility	Short-term financial vision

**T2 diabetes needs a “systems solution”**		

Optimal coaching, participation, and communication	Diagnosis of all relevant processes and predispositions	Patient empowerment
Integration of medical, social, economical, and mental solutions	Goal: regain flexibility in all relevant processes, exploiting diet, lifestyle, medication, and genetics where relevant	Implements regional setting
		Acceptance by accreditation

### The Role of the Healthcare System

The healthcare system will need some adaptations to embrace a lifestyle-based cure program for type 2 diabetes, as prototype for many others to follow (Table 4). Healthcare systems vary by country but some generic issues might be addressed. A better education on diet and lifestyle for medical professionals is needed. A financial system which allows for early investments in lifestyle-based cure (diagnosis, coaching) instead of postponing spending until comorbidities emerge would facilitate implementation. Also, the need for “evidence based accreditation” connected to reimbursement by insurance companies might be a relaxed a bit as the type of lifestyle interventions discussed in this article will never have the economic value to justify “pharma-like” studies. As alternative, the “citizen-science” approach discussed above will slowly substitute the randomized controlled trials now promoted.

### The Economy of Healthcare: From Disease Focused Products to Health-Oriented Services

A sustainable shift toward healthier lifestyle will not be easy to achieve. In fact, until now, neither scientific experiments nor implementation of lifestyle changing programs have demonstrated their efficacy for sustained type 2 diabetes prevention (177). Yet, in treatment of T2D, this has been demonstrated (13, 14). Clearly, individuals themselves are directly responsible for what they eat, how they exercise, etc. However, a complex interplay between many external agents: regulators, industrial sectors, medical professionals, the media, and social networks influence the choices individuals make (178). Making healthier choices are critical for the future health of our bodies and our societies. The multifactorial character of lifestyle change in relation to obesity has been evaluated, taking all biological, economic, and societal aspects into account (15), indicating that no single change will have any major effect on obesity, and a systems approach or multi-domain approach is essential. This might be achieved on a society level but will need a concerted action of all stakeholders and would imply major shifts in economic values, so is unlikely to happen with consensus of the current stakeholders. For example, in the current food supply chain, unhealthy food is cheap and healthy food is expensive, which does not help in making the right food choices for the majority of the population.

The food and nutrition market faces major challenges. The Western world suffers from too much and relatively cheap food with low-nutrient content but high-caloric density, mostly derived from low-cost ingredients like vegetable fat and sugars. This is a trend rapidly adapted by the developing world (179). Food industry finds difficulties in providing scientific evidence that their products are healthy or have added health value (180), which might create added product value. Two key solutions here are the availability of foods with substantiated health benefits and the facilitation of personal healthy food choices, and acknowledging that food can be used as medicine, i.e., removing the artificial legal barrier to connect therapeutic or medical health claims to foods and nutrients (180, 181). These barriers historically make sense but impede the proper use to food in prevention of chronic disease or as therapy to treat lifestyle-related disease.

An essential part in this process is the individual’s self-empowerment in making healthy choices, by having access to reliable and actionable information to one’s personal health status through access to longitudinal personal health data, as described above. In shaping this new reality, self-empowerment needs to be embedded in, and possibly even become the driver of a new healthcare economy based on personal data ownership (171). Development of systems diagnosis with preventive and personalized interventions may create and trigger a series of commercial service-based health industry activities in the area of diagnostics, personal food solutions, food–pharma combinations, health advice systems, etc. Food companies may shift their product portfolio from product branding to product–service combinations (personalized products connected to a diagnostic service), food services may be integrated into a health-based personal portfolio, ICT services will emerge based on a personal Biopassport (interpretation of an individual’s health data and relate this to actionable and understandable nutrition and lifestyle advice). Numerous business opportunities can be envisioned in a health economy which focuses on cure instead of care. These include pharma, food, retail, fitness, artificial intelligence, pharmacy, eHealth, diagnostics, coaching services, and workplace health programs. All of this needs to be developed based on evidence-based science a within adequate regulatory–ethical frameworks. In other words, there is some work to be done.

Motivational coaching, both personal and by using ICT means, can be fully developed and implemented if financed in a healthcare system that appreciates the value of prevention and lifestyle therapy for lifestyle-related diseases. The developments in this area are rapid and promising (182, 183).

## Conclusion and Call to Action

In this article, we provide evidence for the reversibility of insulin resistance and the remission of type 2 diabetes, specifically by diet and lifestyle. Complete cure may be achieved if beta-cell function is still appropriate and complications have not yet occurred. We demonstrate that T2D is a “systems disease” with multiple organs and processes involved and consequently deserves to be treated in a personalized manner, if necessary in a “personalized lifestyle-personalized medicine combination.” Compliance to lifestyle change has been a major obstacle for implementation in health care, but the advancements in behavioral change technologies, eHealth, health literacy, and personal health data valorization now may allow for a switch from a research setting to real-life socioeconomic implementation. Enough arguments and instruments are currently available to implement a lifestyle-based therapy for type 2 diabetes and other food-related lifestyle diseases and to extend this to a prevention and optimal health focused health care. Also, we argue that in doing so, an enormous economic gain will be achieved, which is well able to finance a lifestyle-based prevention and optimal health focused health care and economy. Since stakeholders, losses, and profits in this new economy will substantially differ from the current situation, the current healthcare industry will only slowly transit toward this new situation. Creative ways of implementation thus need to be explored. Ultimately, health data cooperatives may become the basis and drivers for this change, but this will take some time to develop into an economic reality. In the meantime, creative new “ecosystems” need to be explored that combine all necessary instruments for specific type 2 diabetes populations to be really effective. The goal would not only be to demonstrate its therapeutic efficacy but also and possibly more important to demonstrate that a new health economy that provides the services (coaching, ITC, foods, diagnostics, medication, all of these personalized and integrated) can become profitable while significantly reducing the net healthcare costs.

Such ecosystems should preferably be regional, facilitating the simultaneous change of all relevant components if the “change system” to interact. This will allow community building, involvement of local healthcare centers, the local health and lifestyle-related economy, etc. The Chicago based south side diabetics project is a good example (175).

The good message is that many early adopter activities, programs, and movements are already active in this area, covering parts of what is needed. A challenge will be to connect and integrate these into functional and flexible programs that can deliver “tailored systems solutions” depending on the personal and subgroup needs.

The major challenge will be to fund these programs, at least to the point that they become self-sustainable. Here, sustainability does not necessarily imply a profitable service as such, but the identification of new natural funders, i.e., the entities that profit from the new systems. Besides the actual service providers, these can be governments, health insurance, employers, and investors. In other words, only a temporary funding of a transition phase needs to occur, once the architecture of such ecosystems is in place.

## Author Contributions

All authors have contributed significantly, both related to their own scientific discipline and expertise, and to the overall manuscript. First author coordinated the writing and provided the architecture.

## Conflict of Interest Statement

The authors declare that the research was conducted in the absence of any commercial or financial relationships that could be construed as a potential conflict of interest.

## Footnotes

^1^www.gem-measures.org (accessed October 1, 2017).

## References

[1] Heymsfield SB Wadden TA Mechanisms, pathophysiology, and management of obesity. N Engl J Med (2017) 376(3):254–66. 10.1056/NEJMra1514009 28099824

[2] Arsenault BJ Després J-P Cardiovascular disease prevention: lifestyle attenuation of genetic risk. Nat Rev Cardiol (2017) 14:187–8. 10.1038/nrcardio.2017.20 28202900

[3] Perandini LA de Sá-Pinto AL Roschel H Benatti FB Lima FR Bonfá E Exercise as a therapeutic tool to counteract inflammation and clinical symptoms in autoimmune rheumatic diseases. Autoimmun Rev (2012) 12(2):218–24. 10.1016/j.autrev.2012.06.007 22776785

[4] Anand P Kunnumakara AB Sundaram C Harikumar KB Tharakan ST Lai OS Cancer is a preventable disease that requires major lifestyle changes. Pharm Res (2008) 25(9):2097–116. 10.1007/s11095-008-9661-9 18626751 PMC2515569

[5] WCRS. Continuous Update Project (2017). Available from: http://www.wcrf.org/int/research-we-fund/continuous-update-project-cup (Accessed October 1, 2017).

[6] Hood L. A personal journey of discovery: developing technology and changing biology. Annu Rev Anal Chem (Palo Alto Calif) (2008) 1(1):1–43. 10.1146/annurev.anchem.1.031207.113113 20636073

[7] Sagner M McNeil A Puska P Auffray C Price ND Hood L The P4 health spectrum – a predictive, preventive, personalized and participatory continuum for promoting healthspan. Prog Cardiovasc Dis (2017) 59(5):506–21. 10.1016/j.pcad.2016.08.002 27546358

[8] Kish LJ Topol EJ Unpatients – why patients should own their medical data. Nat Biotechnol (2015) 33(9):921–4. 10.1038/nbt.3340 26348958

[9] Bragazzi NL From P0 to P6 medicine, a model of highly participatory, narrative, interactive, and “augmented” medicine: some considerations on Salvatore Iaconesi’s clinical story. Patient Prefer Adherence (2013) 7:353–9. 10.2147/PPA.S38578 23650443 PMC3640773

[10] van Wietmarschen HA Wortelboer HM van der Greef J. Grip on health: a complex systems approach to transform health care. J Eval Clin Pract (2016) 1–9. 10.1111/jep.12679 28032412

[11] Price ND Magis AT Earls JC Glusman G Levy R Lausted C A wellness study of 108 individuals using personal, dense, dynamic data clouds. Nat Biotechnol (2017) 35:747–56. 10.1038/nbt.3870 28714965 PMC5568837

[12] Bloom DE Cafiero ET Jané-Llopis E Abrahams-Gessel S Bloom LR Fathima S The Global Economic Burden of Non-communicable Diseases. Geneva: World Economic Forum (2011).

[13] Knowler WC Barrett-Connor E Fowler SE Hamman RF Lachin JM Walker EA Reduction in the incidence of type 2 diabetes with lifestyle intervention or metformin. N Engl J Med (2002) 346(6):393–403. 10.1056/NEJMoa012512 11832527 PMC1370926

[14] Espeland MA Glick HA Bertoni A Brancati FL Bray GA Clark JM Impact of an intensive lifestyle intervention on use and cost of medical services among overweight and obese adults with type 2 diabetes: the action for health in diabetes. Diabetes Care (2014) 37(9):2548–56. 10.2337/dc14-0093 25147253 PMC4140155

[15] McKinsey Global Institute. Overcoming Obesity: An Initial Economic Analysis Discussion Paper. (2014).

[16] Van Ommen B Wopereis S Next-Generation Nutritional Biomarkers to Guide Better Health Care. In Baetge EE Dhawan A Prentice AM editors. Nestle Nutr Inst Workshop Ser. (2016) 84:25–33. 10.1159/000436949 26764470

[17] Pérez-Martínez P Mikhailidis DP Athyros VG Bullo M Couture P Covas MI Lifestyle recommendations for the prevention and management of metabolic syndrome: an international panel recommendation. Nutr Rev (2017) 75(5):307–26. 10.1093/nutrit/nux014 28521334 PMC5914407

[18] Buse JB Caprio S Cefalu WT Ceriello A Del Prato S Inzucchi SE How do we define cure of diabetes? Diabetes Care (2009) 32(11):2133–5. 10.2337/dc09-9036 19875608 PMC2768219

[19] Gloy VL Briel M Bhatt DL Kashyap SR Schauer PR Mingrone G Bariatric surgery versus non-surgical treatment for obesity: a systematic review and meta-analysis of randomised controlled trials. BMJ (2013) 347:f5934. 10.1136/bmj.f5934 24149519 PMC3806364

[20] Ribaric G Buchwald JN McGlennon TW. Diabetes and weight in comparative studies of bariatric surgery vs conventional medical therapy: a systematic review and meta-analysis. Obes Surg (2014) 24(3):437–55. 10.1007/s11695-013-1160-3 24374842 PMC3916703

[21] Dutia R Brakoniecki K Bunker P Paultre F Homel P Carpentier AC Limited recovery of β-cell function after gastric bypass despite clinical diabetes remission. Diabetes (2014) 63(4):1214–23. 10.2337/db13-1176 24296713 PMC3964502

[22] Chen L Pei J-H Kuang J Chen H-M Chen Z Li Z-W Effect of lifestyle intervention in patients with type 2 diabetes: a meta-analysis. Metabolism (2015) 64(2):338–47. 10.1016/j.metabol.2014.10.018 25467842

[23] Lim EL Hollingsworth KG Aribisala BS Chen MJ Mathers JC Taylor R. Reversal of type 2 diabetes: normalisation of beta cell function in association with decreased pancreas and liver triacylglycerol. Diabetologia (2011) 54(10):2506–14. 10.1007/s00125-011-2204-7 21656330 PMC3168743

[24] Cheng C-W Villani V Buono R Sneddon JB Perin L Longo Correspondence VD Fasting-mimicking diet promotes Ngn3-driven B-cell regeneration to reverse diabetes in brief fasting-mimicking diet promotes Ngn3-driven B-cell regeneration to reverse diabetes. Cell (2017) 168(5):775–88.e12. 10.1016/j.cell.2017.01.040 28235195 PMC5357144

[25] Tuomi T Miettinen PJ Hakaste L Groop L Atypical forms of diabetes. Endotext. (2000). Available from: http://www.ncbi.nlm.nih.gov/pubmed/25905351

[26] Segrè AV Wei N DIAGRAM Consortium, MAGIC Investigators Altshuler D Florez JC. Pathways targeted by antidiabetes drugs are enriched for multiple genes associated with type 2 diabetes risk. Diabetes (2015) 64(4):1470–83. 10.2337/db14-0703 25368101 PMC4375079

[27] Kardinaal AF van Erk MJ Dutman AE Stroeve JH van de Steeg E Bijlsma S Quantifying phenotypic flexibility as the response to a high-fat challenge test in different states of metabolic health. FASEB J (2015) 29(11):4600–13. 10.1096/fj.14-269852 26198450

[28] van Ommen B van der Greef J Ordovas JM Daniel H. Phenotypic flexibility as key factor in the human nutrition and health relationship. Genes Nutr (2014) 9(5):423. 10.1007/s12263-014-0423-5 25106484 PMC4172643

[29] van Ommen B van den Broek T de Hoogh I van Erk M van Someren E Rouhani-Rankouhi T Systems biology of personalized nutrition. Nutr Rev (2017) 75(8):579–99. 10.1093/nutrit/nux029 28969366 PMC5914356

[30] Stroeve JHM van Wietmarschen H Kremer BHA van Ommen B Wopereis S. Phenotypic flexibility as a measure of health: the optimal nutritional stress response test. Genes Nutr (2015) 10(3):459. 10.1007/s12263-015-0459-1 25896408 PMC4404421

[31] van der Greef J Hankemeier T McBurney RN. Metabolomics-based systems biology and personalized medicine: moving towards n = 1 clinical trials? Pharmacogenomics (2006) 7(7):1087–94. 10.2217/14622416.7.7.1087 17054418

[32] Liss KHH Finck BN PPARs and nonalcoholic fatty liver disease. Biochimie (2016) 136:65–74. 10.1016/j.biochi.2016.11.009 27916647 PMC5380579

[33] Sherriff JL OSullivan TA Properzi C Oddo J-L Adams LA. Choline, its potential role in nonalcoholic fatty liver disease, and the case for human and bacterial genes. Adv Nutr (2016) 7(1):5–13. 10.3945/an.114.007955 26773011 PMC4717871

[34] Malaguarnera M Gargante MP Russo C Antic T Vacante M Malaguarnera M l-carnitine supplementation to diet: a new tool in treatment of nonalcoholic steatohepatitis – a randomized and controlled clinical trial. Am J Gastroenterol (2010) 105(6):1338–45. 10.1038/ajg.2009.719 20068559

[35] de Wit NJ Afman LA Mensink M Müller M. Phenotyping the effect of diet on non-alcoholic fatty liver disease. J Hepatol (2012) 57(6):1370–3. 10.1016/j.jhep.2012.07.003 22796155

[36] Blanco-Rojo R Alcala-Diaz JF Wopereis S Perez-Martinez P Quintana-Navarro GM Marin C The insulin resistance phenotype (muscle or liver) interacts with the type of diet to determine changes in disposition index after 2 years of intervention: the CORDIOPREV-DIAB randomised clinical trial. Diabetologia (2015) 59(1):67–76. 10.1007/s00125-015-3776-4 26474775

[37] van Ommen B Keijer J Heil SG Kaput JJJ. Challenging homeostasis to define biomarkers for nutrition related health. Mol Nutr Food Res (2009) 53(7):795–804. 10.1002/mnfr.200800390 19517455

[38] Wopereis S Stroeve JHM Stafleu A Bakker GCM Burggraaf J van Erk MJ Multi-parameter comparison of a standardized mixed meal tolerance test in healthy and type 2 diabetic subjects: the PhenFlex challenge. Genes Nutr (2017) 12(1):21. 10.1186/s12263-017-0570-6 28861127 PMC5576306

[39] Pellis L Erk MJ Ommen B Bakker GCM Hendriks HFJ Cnubben NHP Plasma metabolomics and proteomics profiling after a postprandial challenge reveal subtle diet effects on human metabolic status. Metabolomics (2011) 8(2):347–59. 10.1007/s11306-011-0320-5 22448156 PMC3291817

[40] van den Broek TJ Kremer BHA Marcondes Rezende M Hoevenaars FPM Weber P Hoeller U The impact of micronutrient status on health: correlation network analysis to understand the role of micronutrients in metabolic-inflammatory processes regulating homeostasis and phenotypic flexibility. Genes Nutr (2017) 12(1):5. 10.1186/s12263-017-0553-7 28194237 PMC5299688

[41] Krug S Kastenmüller G Stückler F Rist MJ Skurk T Sailer M The dynamic range of the human metabolome revealed by challenges. FASEB J (2012) 26:2607–19. 10.1096/fj.11-198093 22426117

[42] Young-Hyman D De Groot M Hill-Briggs F Gonzalez JS Hood K Peyrot M Psychosocial care for people with diabetes: a position statement of the American Diabetes Association. Diabetes Care (2016) 39(12):2126–40. 10.2337/dc16-2053 27879358 PMC5127231

[43] Vasan RS Benjamin EJ The future of cardiovascular epidemiology. Circulation (2016) 133(25):2626–33. 10.1161/CIRCULATIONAHA.116.023528 27324358 PMC4974092

[44] Barabási A-L Network medicine – from obesity to the “diseasome”. N Engl J Med (2007) 357(4):404–7. 10.1056/NEJMe078114 17652657

[45] Singer M Introduction to Syndemics: A Critical Systems Approach to Public and Community Health. San Francisco: Wiley Online Library (2009).

[46] Wadden TA. Eight-year weight losses with an intensive lifestyle intervention: the look AHEAD study. Obesity (2014) 22(1):5–13. 10.1002/oby.20662 24307184 PMC3904491

[47] Franz MJ Boucher JL Evert AB. Evidence-based diabetes nutrition therapy recommendations are effective: the key is individualization. Diabetes Metab Syndr Obes (2014) 7:65–72. 10.2147/DMSO.S45140 24591844 PMC3938438

[48] Van Gaal L Scheen A. Weight management in type 2 diabetes: current and emerging approaches to treatment. Diabetes Care (2015) 38(6):1161–72. 10.2337/dc14-1630 25998297

[49] Steven S Hollingsworth KG Small PK Woodcock SA Pucci A Aribisala B Weight loss decreases excess pancreatic triacylglycerol specifically in type 2 diabetes. Diabetes Care (2016) 39(1):158–65. 10.2337/dc15-0750 26628414

[50] Steven S Hollingsworth KG Al-Mrabeh A Avery L Aribisala B Caslake M Very-low-calorie diet and 6 months of weight stability in type 2 diabetes: pathophysiologic changes in responders and nonresponders. Diabetes Care (2016) 39(5):808–15. 10.2337/dc15-1942 27002059

[51] Steven S Lim EL Taylor R. Population response to information on reversibility of type 2 diabetes. Diabet Med (2013) 30(4):e135–8. 10.1111/dme.12116 23320491

[52] Lean ME Leslie WS Barnes AC Brosnahan N Thom G Mccombie L Primary care-led weight management for remission of type 2 diabetes (DiRECT): an open-label, cluster-randomised trial. Lancet (2017) 6736(17):1–11. 10.1016/S0140-6736(17)33102-1 29221645

[53] Varady KA Hudak CS Hellerstein MK. Modified alternate-day fasting and cardioprotection: relation to adipose tissue dynamics and dietary fat intake. Metabolism (2009) 58(6):803–11. 10.1016/j.metabol.2009.01.018 19375762

[54] Carter S Clifton PM Keogh JB. The effects of intermittent compared to continuous energy restriction on glycaemic control in type 2 diabetes; a pragmatic pilot trial. Diabetes Res Clin Pract (2016) 122:106–12. 10.1016/j.diabres.2016.10.010 27833048

[55] Ash S Reeves MM Yeo S Morrison G Carey D Capra S. Effect of intensive dietetic interventions on weight and glycaemic control in overweight men with type II diabetes: a randomised trial. Int J Obes (2003) 27(7):797. 10.1038/sj.ijo.0802295 12821964

[56] Tinsley GM La Bounty PM. Effects of intermittent fasting on body composition and clinical health markers in humans. Nutr Rev (2015) 73(10):661–74. 10.1093/nutrit/nuv041 26374764

[57] Halberg N Henriksen M Söderhamn N Stallknecht B Ploug T Schjerling P Effect of intermittent fasting and refeeding on insulin action in healthy men. J Appl Physiol (2005) 99(6):2128. 10.1152/japplphysiol.00683.2005 16051710

[58] Anson RM Guo Z de Cabo R Iyun T Rios M Hagepanos A Intermittent fasting dissociates beneficial effects of dietary restriction on glucose metabolism and neuronal resistance to injury from calorie intake. Proc Natl Acad Sci U S A (2003) 100(10):6216–20. 10.1073/pnas.1035720100 12724520 PMC156352

[59] Longo VD Mattson MP. Fasting: molecular mechanisms and clinical applications. Cell Metab (2014) 19(2):181–92. 10.1016/j.cmet.2013.12.008 24440038 PMC3946160

[60] Mirzaei H Suarez JA Longo VD. Protein and amino acid restriction, aging and disease: from yeast to humans. Trends Endocrinol Metab (2014) 25(11):558–66. 10.1016/j.tem.2014.07.002 25153840 PMC4254277

[61] Brandhorst S Choi IY Wei M Cheng CW Sedrakyan S Navarrete G A periodic diet that mimics fasting promotes multi-system regeneration, enhanced cognitive performance, and healthspan. Cell Metab (2015) 22(1):86–99. 10.1016/j.cmet.2015.05.012 26094889 PMC4509734

[62] Wei M Brandhorst S Shelehchi M Mirzaei H Cheng CW Budniak J Fasting-mimicking diet and markers/risk factors for aging, diabetes, cancer, and cardiovascular disease. Sci Transl Med (2017) 9(377):eaai8700. 10.1126/scitranslmed.aai8700 28202779 PMC6816332

[63] Harvie MN Pegington M Mattson MP Frystyk J Dillon B Cuzick J The effects of intermittent or continuous restriction on weight loss and metabolic disease risk markers: a randomised trial in young overweight women. Int J Obes (2011) 35(5):714–27. 10.1038/ijo.2010.171 PMC3017674 20921964

[64] Mattson MP Longo VD Harvie M Impact of intermittent fasting on health and disease processes. Ageing Res Rev (2016) 39:46–58. 10.1016/j.arr.2016.10.005 27810402 PMC5411330

[65] Heilbronn LK Smith SR Martin CK Anton SD Ravussin E. Alternate-day fasting in nonobese subjects: effects on body weight, body composition, and energy metabolism. Am J Clin Nutr (2005) 81:69–73. 15640462 10.1093/ajcn/81.1.69

[66] Carlson O Martin B Stote KS Golden E Maudsley S Najjar SS Impact of reduced meal frequency without caloric restriction on glucose regulation in healthy, normal-weight middle-aged men and women. Metabolism (2007) 56(12):1729–34. 10.1016/j.metabol.2007.07.018 17998028 PMC2121099

[67] Tay J Luscombe-Marsh ND Thompson CH Noakes M Buckley JD Wittert GA A very low-carbohydrate, low-saturated fat diet for type 2 diabetes management: a randomized trial. Diabetes Care (2014) 37(11):2909–18. 10.2337/dc14-0845 25071075

[68] Westman EC Yancy WS Mavropoulos JC Marquart M McDuffie JR. The effect of a low-carbohydrate, ketogenic diet versus a low-glycemic index diet on glycemic control in type 2 diabetes mellitus. Nutr Metab (Lond) (2008) 5(1):36. 10.1186/1743-7075-5-36 19099589 PMC2633336

[69] Kosinski C Jornayvaz F. Effects of ketogenic diets on cardiovascular risk factors: evidence from animal and human studies. Nutrients (2017) 9(6):517. 10.3390/nu9050517 28534852 PMC5452247

[70] Mayer SB Jeffreys AS Olsen MK McDuffie JR Feinglos MN Yancy WS. Two diets with different haemoglobin A1c and antiglycaemic medication effects despite similar weight loss in type 2 diabetes. Diabetes Obes Metab (2014) 16(1):90–3. 10.1111/dom.12191 23911112 PMC3867584

[71] Samaha FF Iqbal N Seshadri P Chicano KL Daily DA McGrory J A low-carbohydrate as compared with a low-fat diet in severe obesity. N Engl J Med (2003) 348(21):2074–81. 10.1056/NEJMoa022637 12761364

[72] Snorgaard O Poulsen GM Andersen HK Astrup A. Systematic review and meta-analysis of dietary carbohydrate restriction in patients with type 2 diabetes. BMJ Open Diabetes Res Care (2017) 5(1):e000354. 10.1136/bmjdrc-2016-000354 28316796 PMC5337734

[73] Imamura F Micha R Wu JHYJ de Oliveira Otto MC Otite FO Abioye AI Effects of saturated fat, polyunsaturated fat, monounsaturated fat, and carbohydrate on glucose-insulin homeostasis: a systematic review and meta-analysis of randomised controlled feeding trials. PLoS Med (2016) 13(7):e1002087. 10.1371/journal.pmed.1002087 27434027 PMC4951141

[74] Heer M Egert S. Nutrients other than carbohydrates: their effects on glucose homeostasis in humans. Diabetes Metab Res Rev (2015) 31(1):14–35. 10.1002/dmrr.2533 24510463

[75] Graf S Egert S Heer M. Effects of whey protein supplements on metabolism: evidence from human intervention studies. Curr Opin Clin Nutr Metab Care (2011) 14(6):569–80. 10.1097/MCO.0b013e32834b89da 21912246

[76] Gunnerud UJ Östman EM Björck IME. Effects of whey proteins on glycaemia and insulinaemia to an oral glucose load in healthy adults; a dose-response study. Eur J Clin Nutr (2013) 67(7):749. 10.1038/ejcn.2013.88 23632747

[77] Smith GI Yoshino J Kelly SC Reeds DN Okunade A Patterson BW High-protein intake during weight loss therapy eliminates the weight-loss-induced improvement in insulin action in obese postmenopausal women. Cell Rep (2016) 17(3):849–61. 10.1016/j.celrep.2016.09.047 27732859 PMC5113728

[78] Linn T Santosa B Grönemeyer D Aygen S Scholz N Busch M Effect of long-term dietary protein intake on glucose metabolism in humans. Diabetologia (2000) 43(10):1257–65. 10.1007/s001250051521 11079744

[79] Weickert MO. What dietary modification best improves insulin sensitivity and why? Clin Endocrinol (Oxf) (2012) 77(4):508–12. 10.1111/j.1365-2265.2012.04450.x 22640465

[80] Chabosseau P Rutter GA Zinc and diabetes. Arch Biochem Biophys (2016) 611:79–85. 10.1016/j.abb.2016.05.022 27262257

[81] Berridge MJ Vitamin D deficiency and diabetes. Biochem J (2017) 474(8):1321–32. 10.1042/BCJ20170042 28341729

[82] Mingrone G Carnitine in type 2 diabetes. Ann N Y Acad Sci (2004) 1033:99–107. 10.1196/annals.1320.009 15591007

[83] Malaguarnera M Vacante M Giordano M Pennisi G Bella R Rampello L Oral acetyl-l-carnitine therapy reduces fatigue in overt hepatic encephalopathy: a randomized, double-blind, placebo-controlled study. Am J Clin Nutr (2011) 93(4):799–808. 10.3945/ajcn.110.007393 21310833

[84] Dongiovanni P Lanti C Riso P Valenti L Nutritional therapy for non-alcoholic fatty liver disease. J Nutr Biochem (2015) 29:1–11. 10.1016/j.jnutbio.2015.08.024 26895659

[85] Merone L McDermott R. Nutritional anti-inflammatories in the treatment and prevention of type 2 diabetes mellitus and the metabolic syndrome. Diabetes Res Clin Pract (2017) 127:238–53. 10.1016/j.diabres.2017.02.019 28402903

[86] Abbott KA Burrows TL Thota RN Acharya S Garg ML Do ω-3 PUFAs affect insulin resistance in a sex-specific manner? A systematic review and meta-analysis of randomized controlled trials. Am J Clin Nutr (2016) 104(5):1470–84. 10.3945/ajcn.116.138172 27680989

[87] Chimienti F. Zinc, pancreatic islet cell function and diabetes: new insights into an old story. Nutr Res Rev (2013) 26(1):1–11. 10.1017/S0954422412000212 23286442

[88] Veronese N Watutantrige-Fernando S Luchini C Solmi M Sartore G Sergi G Effect of magnesium supplementation on glucose metabolism in people with or at risk of diabetes: a systematic review and meta-analysis of double-blind randomized controlled trials. Eur J Clin Nutr (2016) 70:1354–9. 10.1038/ejcn.2016.154 27530471

[89] Garbossa SG Folli F. Vitamin D, sub-inflammation and insulin resistance. A window on a potential role for the interaction between bone and glucose metabolism. Rev Endocr Metab Disord (2017) 18(2):243–58. 10.1007/s11154-017-9423-2 28409320

[90] DeFronzo RA. Insulin resistance, lipotoxicity, type 2 diabetes and atherosclerosis: the missing links. The Claude Bernard Lecture 2009. Diabetologia (2010) 53(7):1270–87. 10.1007/s00125-010-1684-1 20361178 PMC2877338

[91] Abdul-Ghani MA Contributions of cell dysfunction and insulin resistance to the pathogenesis of impaired glucose tolerance and impaired fasting glucose. Diabetes Care (2006) 29(5):1130–9. 10.2337/dc05-2179 16644654

[92] Snel M Jonker JT Schoones J Lamb H de Roos A Pijl H Ectopic fat and insulin resistance: pathophysiology and effect of diet and lifestyle interventions. Int J Endocrinol (2012) 2012:983814. 10.1155/2012/983814 22675355 PMC3366269

[93] Dela F von Linstow ME Mikines KJ Galbo H. Physical training may enhance beta-cell function in type 2 diabetes. Am J Physiol Endocrinol Metab (2004) 287(5):E1024–31. 10.1152/ajpendo.00056.2004 15251867

[94] Burns N Finucane FM Hatunic M Gilman M Murphy M Gasparro D Early-onset type 2 diabetes in obese white subjects is characterised by a marked defect in beta cell insulin secretion, severe insulin resistance and a lack of response to aerobic exercise training. Diabetologia (2007) 50(7):1500–8. 10.1007/s00125-007-0655-7 17393133

[95] Snel M Gastaldelli A Ouwens DM Hesselink MKC Schaart G Buzzigoli E Effects of adding exercise to a 16-week very low-calorie diet in obese, insulin-dependent type 2 diabetes mellitus patients. J Clin Endocrinol Metab (2012) 97(7):2512–20. 10.1210/jc.2011-3178 22569236

[96] Goodpaster BH Sparks LM. Metabolic flexibility in health and disease. Cell Metab (2017) 25(5):1027–36. 10.1016/j.cmet.2017.04.015 28467922 PMC5513193

[97] Houston M The role of nutraceutical supplements in the treatment of hypertension. J Clin Hypertens (2012) 14:121–32. 10.1111/j.1751-7176.2011.00576.x PMC9645405 22277145

[98] Pedersen BK Saltin B. Evidence for prescribing exercise as therapy in chronic disease. Scand J Med Sci Sports (2006) 16(Suppl 1):3–63. 10.1111/j.1600-0838.2006.00520.x 16451303

[99] Pedersen BK Saltin B. Exercise as medicine – evidence for prescribing exercise as therapy in 26 different chronic diseases. Scand J Med Sci Sports (2015) 25:1–72. 10.1111/sms.12581 26606383

[100] Asano RY Sales MM Browne RA Moraes JF Coelho Junior HJ Moraes MR Acute effects of physical exercise in type 2 diabetes: a review. World J Diabetes (2014) 5(5):659–65. 10.4239/wjd.v5.i5.659 25317243 PMC4138589

[101] Orci LA Gariani K Oldani G Delaune V Morel P Toso C. Exercise-based interventions for nonalcoholic fatty liver disease: a meta-analysis and meta-regression. Clin Gastroenterol Hepatol (2016) 14(10):1398–411. 10.1016/j.cgh.2016.04.036 27155553

[102] Cassidy S Thoma C Hallsworth K Parikh J Hollingsworth KG Taylor R High intensity intermittent exercise improves cardiac structure and function and reduces liver fat in patients with type 2 diabetes: a randomised controlled trial. Diabetologia (2016) 59(1):56–66. 10.1007/s00125-015-3741-2 26350611 PMC4670457

[103] Schiavon M Hinshaw L Mallad A Dalla Man C Sparacino G Johnson M Postprandial glucose fluxes and insulin sensitivity during exercise: a study in healthy individuals. Am J Physiol Endocrinol Metab (2013) 305(4):E557–66. 10.1152/ajpendo.00182.2013 23820621 PMC3891224

[104] Pedersen BK The diseasome of physical inactivity – and the role of myokines in muscle-fat cross talk. J Physiol (2009) 587(23):5559–68. 10.1113/jphysiol.2009.179515 19752112 PMC2805368

[105] Fletcher E Leech R McNaughton SA Dunstan DW Lacy KE Salmon J. Is the relationship between sedentary behaviour and cardiometabolic health in adolescents independent of dietary intake? A systematic review. Obes Rev (2015) 16(9):795–805. 10.1111/obr.12302 26098509 PMC4657480

[106] Solomon TPJ Thyfault JP. Type 2 diabetes sits in a chair. Diabetes Obes Metab (2013) 15(11):987–92. 10.1111/dom.12105 23551885

[107] Sabag A Way KL Keating SE Sultana RN O’Connor HT Baker MK Exercise and ectopic fat in type 2 diabetes: a systematic review and meta-analysis. Diabetes Metab (2017) 43(3):195–210. 10.1016/j.diabet.2016.12.006 28162956

[108] Pedersen BK. Anti-inflammatory effects of exercise: role in diabetes and cardiovascular disease. Eur J Clin Invest (2017) 42:105–17. 10.1111/eci.12781 28722106

[109] Hesselink MKC Schrauwen-Hinderling V Schrauwen P. Skeletal muscle mitochondria as a target to prevent or treat type 2 diabetes mellitus. Nat Rev Endocrinol (2016) 12(11):633–45. 10.1038/nrendo.2016.104 27448057

[110] Anderson JW Ward K. High-carbohydrate, high-fiber diets for insulin-treated men with diabetes mellitus. Am J Clin Nutr (1979) 32:2312–21. 495550 10.1093/ajcn/32.11.2312

[111] Reusch JEB Manson JE Management of type 2 diabetes in 2017. JAMA (2017) 317(10):1015 10.1001/jama.2017.0241 28249081 PMC5894353

[112] Wang Z York NW Nichols CG Remedi MS Pancreatic beta-cell dedifferentiation in diabetes and redifferentiation following insulin therapy. Cell Metab (2014) 19(5):872–82. 10.1016/j.cmet.2014.03.010 24746806 PMC4067979

[113] Rothman AJ Baldwin AS Hertel AW Self-regulation and behavior change: disentangling behavioral initiation and behavioral maintenance. In: Vohs KD Baumeister RF, editors. Handbook of Self-Regulation. London: The Guilford Press (2004). p. 130–50.

[114] Janssen V De Gucht V Dusseldorp E Maes S. Lifestyle modification programmes for patients with coronary heart disease: a systematic review and meta-analysis of randomized controlled trials. Eur J Prev Cardiol (2013) 20(4):620–40. 10.1177/2047487312462824 23022703

[115] Sniehotta FF Scholz U Schwarzer R Fuhrmann B Kiwus U Völler H. Long-term effects of two psychological interventions on physical exercise and self-regulation following coronary rehabilitation. Int J Behav Med (2005) 12(4):244–55. 10.1207/s15327558ijbm1204_5 16262543

[116] Ryan RM Deci EL. Self-determination theory and the facilitation of intrinsic motivation, social development, and well-being. Am Psychol (2000) 55(1):68–78. 10.1037/0003-066X.55.1.68 11392867

[117] Michie S Wood CE Johnston M Abraham C Francis JJ Hardeman W Behaviour change techniques: the development and evaluation of a taxonomic method for reporting and describing behaviour change interventions (a suite of five studies involving consensus methods, randomised controlled trials and analysis of qualitative data). Health Technol Assess (Rockv) (2015) 19(99):1–187. 10.3310/hta19990 PMC4781650 26616119

[118] Dusseldorp E van Genugten L van Buuren S Verheijden MW van Empelen P. Combinations of techniques that effectively change health behavior: evidence from meta-CART analysis. Health Psychol (2014) 33(12):1530–40. 10.1037/hea0000018 24274802

[119] Ontario Health Department. Community-based care for the management of type 2 diabetes: an evidence-based analysis. Ont Health Technol Assess Ser (2009) 9:1–40. PMC3377524 23074528

[120] Fjeldsoe B Neuhaus M Winkler E Eakin E. Systematic review of maintenance of behavior change following physical activity and dietary interventions. Health Psychol (2011) 30(1):99–109. 10.1037/a0021974 21299298

[121] Avery L Flynn D Dombrowski SU van Wersch A Sniehotta FF Trenell MI Successful behavioural strategies to increase physical activity and improve glucose control in adults with type 2 diabetes. Diabet Med (2015) 32(8):1058–62. 10.1111/dme.12738 25764343 PMC6680111

[122] Kwasnicka D Dombrowski SU White M Sniehotta F. Theoretical explanations for maintenance of behaviour change: a systematic review of behaviour theories. Health Psychol Rev (2016) 10(3):277–96. 10.1080/17437199.2016.1151372 26854092 PMC4975085

[123] Michie S Richardson M Johnston M Abraham C Francis J Hardeman W The behavior change technique taxonomy (v1) of 93 hierarchically clustered techniques: building an international consensus for the reporting of behavior change interventions. Ann Behav Med (2013) 46(1):81–95. 10.1007/s12160-013-9486-6 23512568

[124] Giles EL Robalino S McColl E Sniehotta FF Adams J The effectiveness of financial incentives for health behaviour change: systematic review and meta-analysis. PLoS One (2014) 9(3):e90347 10.1371/journal.pone.0090347 24618584 PMC3949711

[125] Mohr S Liew C-C. The peripheral-blood transcriptome: new insights into disease and risk assessment. Trends Mol Med (2007) 13(10):422–32. 10.1016/j.molmed.2007.08.003 17919976

[126] Charlier N Zupancic N Fieuws S Denhaerynck K Zaman B Moons P. Serious games for improving knowledge and self-management in young people with chronic conditions: a systematic review and meta-analysis. J Am Med Inform Assoc (2016) 23(1):230–9. 10.1093/jamia/ocv100 26186934 PMC7814922

[127] DeSmet A Van Ryckeghem D Compernolle S Baranowski T Thompson D Crombez G A meta-analysis of serious digital games for healthy lifestyle promotion. Prev Med (2014) 69:95–107. 10.1016/j.ypmed.2014.08.026 25172024 PMC4403732

[128] Christensen J Valentiner LS Petersen RJ Langberg H. The effect of game-based interventions in rehabilitation of diabetics: a systematic review and meta-analysis. Telemed J E Health (2016) 22(10):789–97. 10.1089/tmj.2015.0165 27042966

[129] Höchsmann C Schüpbach M Schmidt-Trucksäss A. Effects of exergaming on physical activity in overweight individuals. Sports Med (2016) 46(6):845–60. 10.1007/s40279-015-0455-z 26712512

[130] Höchsmann C Walz SP Schäfer J Holopainen J Hanssen H Schmidt-Trucksäss A Mobile exergaming for health-effects of a serious game application for smartphones on physical activity and exercise adherence in type 2 diabetes mellitus – study protocol for a randomized controlled trial. Trials (2017) 18(1):103 10.1186/s13063-017-1853-3 28264717 PMC5339965

[131] Elvins R Green J. The conceptualization and measurement of therapeutic alliance: an empirical review. Clin Psychol Rev (2008) 28(7):1167–87. 10.1016/j.cpr.2008.04.002 18538907

[132] Clarke J Proudfoot J Whitton A Birch M-R Boyd M Parker G Therapeutic alliance with a fully automated mobile phone and web-based intervention: secondary analysis of a randomized controlled trial. JMIR Ment Health (2016) 3(1):e10. 10.2196/mental.4656 26917096 PMC4786687

[133] van Beugen S Ferwerda M Spillekom-van Koulil S Smit JV Zeeuwen-Franssen MEJ Kroft EBM Tailored therapist-guided Internet-based cognitive behavioral treatment for psoriasis: a randomized controlled trial. Psychother Psychosom (2016) 85(5):297–307. 10.1159/000447267 27508937

[134] Bailey SC Brega AG Crutchfield TM Elasy T Herr H Kaphingst K Update on health literacy and diabetes. Diabetes Educ (2014) 40(5):581–604. 10.1177/0145721714540220 24947871 PMC4174500

[135] Watts SA Stevenson C Adams M Improving health literacy in patients with diabetes. Nursing (Lond) (2017) 47(1):24–31. 10.1097/01.NURSE.0000510739.60928.a9 27922896

[136] Mendenhall E Kohrt BA Norris SA Ndetei D Prabhakaran D. Non-communicable disease syndemics: poverty, depression, and diabetes among low-income populations. Lancet (2017) 389(10072):951–63. 10.1016/S0140-6736(17)30402-6 28271846 PMC5491333

[137] Blankenship KM Friedman SR Dworkin S Mantell JE. Structural interventions: concepts, challenges and opportunities for research. J Urban Health (2006) 83(1):59–72. 10.1007/s11524-005-9007-4 16736355 PMC1473169

[138] Liburd LC Jack L Williams S Tucker P. Intervening on the social determinants of cardiovascular disease and diabetes. Am J Prev Med (2005) 29(5):18–24. 10.1016/j.amepre.2005.07.013 16389121

[139] van Gemert-Pijnen JE Nijland N van Limburg M Ossebaard HC Kelders SM Eysenbach G A holistic framework to improve the uptake and impact of eHealth technologies. J Med Internet Res (2011) 13(4):e111. 10.2196/jmir.1672 22155738 PMC3278097

[140] van Beugen S Ferwerda M Hoeve D Rovers MM Spillekom-van Koulil S van Middendorp H Internet-based cognitive behavioral therapy for patients with chronic somatic conditions: a meta-analytic review. J Med Internet Res (2014) 16(3):e88. 10.2196/jmir.2777 24675372 PMC4004147

[141] Rollo ME Aguiar EJ Williams RL Wynne K Kriss M Callister R eHealth technologies to support nutrition and physical activity behaviors in diabetes. Diabetes Metab Syndr Obes (2016) 9:381–90. 10.2147/DMSO.S95247 27853384 PMC5104301

[142] Lieber BA Taylor B Appelboom G Prasad K Bruce S Yang A Meta-analysis of telemonitoring to improve HbA1c levels: promise for stroke survivors. J Clin Neurosci (2015) 22(5):807–11. 10.1016/j.jocn.2014.11.009 25791996

[143] Mushcab H Kernohan WG Wallace J Martin S. Web-based remote monitoring systems for self-managing type 2 diabetes: a systematic review. Diabetes Technol Ther (2015) 17(7):498–509. 10.1089/dia.2014.0296 25830528

[144] Bashshur RL Shannon GW Smith BR Woodward MA. The empirical evidence for the telemedicine intervention in diabetes management. Telemed J E Health (2015) 21(5):321–54. 10.1089/tmj.2015.0029 25806910 PMC4432488

[145] Hood M Wilson R Corsica J Bradley L Chirinos D Vivo A. What do we know about mobile applications for diabetes self-management? A review of reviews. J Behav Med (2016) 39(6):981–94. 10.1007/s10865-016-9765-3 27412774

[146] Whitehead L Seaton P. The effectiveness of self-management mobile phone and tablet apps in long-term condition management: a systematic review. J Med Internet Res (2016) 18(5):e97. 10.2196/jmir.4883 27185295 PMC4886099

[147] Campbell R Ash J. An evaluation of five bedside information products using a user-centered, task-oriented approach. J Med Libr Assoc (2006) 94(4):435–41, e206–e207. 17082836 PMC1629448

[148] Wishart DS Knox C Guo AC Eisner R Young N Gautam B HMDB: a knowledgebase for the human metabolome. Nucleic Acids Res (2009) 37(Suppl_1):D603–10. 10.1093/nar/gkn810 18953024 PMC2686599

[149] Chomutare T Fernandez-Luque L Årsand E Hartvigsen G. Features of mobile diabetes applications: review of the literature and analysis of current applications compared against evidence-based guidelines. J Med Internet Res (2011) 13(3):e65. 10.2196/jmir.1874 21979293 PMC3222161

[150] Wu Y Yao X Vespasiani G Nicolucci A Dong Y Kwong J Mobile app-based interventions to support diabetes self-management: a systematic review of randomized controlled trials to identify functions associated with glycemic efficacy. JMIR Mhealth Uhealth (2017) 5(3):e35. 10.2196/mhealth.6522 28292740 PMC5373677

[151] American Diabetes Association. 2. Classification and diagnosis of diabetes. Diabetes Care (2016) 39(Suppl 1):S13–22. 10.2337/dc16-S005 26696675

[152] Sepah SC Jiang L Peters AL. Long-term outcomes of a web-based diabetes prevention program: 2-year results of a single-arm longitudinal study. J Med Internet Res (2015) 17(4):e92. 10.2196/jmir.4052 25863515 PMC4409647

[153] Gallwitz B Implications of postprandial glucose and weight control in people with type 2 diabetes: understanding and implementing the international diabetes federation guidelines. Diabetes Care (2009) 32(Suppl_2):S322–5. 10.2337/dc09-S331 19875573 PMC2811482

[154] Webb TL Sniehotta FF Michie S. Using theories of behaviour change to inform interventions for addictive behaviours. Addiction (2010) 105(11):1879–92. 10.1111/j.1360-0443.2010.03028.x 20670346

[155] Kreuter MW Lukwago SN Bucholtz DC Clark EM Sanders-Thompson V. Achieving cultural appropriateness in health promotion programs: targeted and tailored approaches. Health Educ Behav (2003) 30(2):133–46. 10.1177/1090198102251021 12693519

[156] Krebs P Prochaska JO Rossi JS. A meta-analysis of computer-tailored interventions for health behavior change. Prev Med (2010) 51(3–4):214–21. 10.1016/j.ypmed.2010.06.004 20558196 PMC2939185

[157] Boh B Lemmens LHJM Jansen A Nederkoorn C Kerkhofs V Spanakis G An ecological momentary intervention for weight loss and healthy eating via smartphone and Internet: study protocol for a randomised controlled trial. Trials (2016) 17(1):154. 10.1186/s13063-016-1280-x 27000058 PMC4802730

[158] Griffith M Siminerio L Payne T Krall J. A shared decision-making approach to telemedicine: engaging rural patients in glycemic management. J Clin Med (2016) 5(11):103. 10.3390/jcm5110103 27869655 PMC5126800

[159] Harter M Dirmaier J Dwinger S Kriston L Herbarth L Siegmund-Schultze E Effectiveness of telephone-based health coaching for patients with chronic conditions: a randomised controlled trial. PLoS One (2016) 11(9):e0161269. 10.1371/journal.pone.0161269 27632360 PMC5025178

[160] Mohr DC Cheung K Schueller SM Brown CH Duan N. Continuous evaluation of evolving behavioral intervention technologies. Am J Prev Med (2013) 45(4):517–23. 10.1016/j.amepre.2013.06.006 24050429 PMC3828034

[161] van Ommen B The nutrition researcher cohort: toward a new generation of nutrition research and health optimization. Genes Nutr (2013) 8(4):343–4. 10.1007/s12263-013-0348-4 23744007 PMC3689895

[162] Vayena E Tasioulas J The ethics of participant-led biomedical research. Nat Biotechnol (2013) 31(9):786–7. 10.1038/nbt.2692 24022150

[163] Collins FS Varmus H. A new initiative on precision medicine. N Engl J Med (2015) 372(9):793–5. 10.1056/NEJMp1500523 25635347 PMC5101938

[164] Kohn MS Sun J Knoop S Shabo A Carmeli B Sow D IBM’s health analytics and clinical decision support. Yearb Med Inform (2014) 9(1):154–62. 10.15265/IY-2014-0002 25123736 PMC4287097

[165] Tkachenko N Chotvijit S Gupta N Bradley E Gilks C Guo W Google trends can improve surveillance of type 2 diabetes. Sci Rep (2017) 7(1):4993. 10.1038/s41598-017-05091-9 28694479 PMC5504026

[166] Ayers JW Althouse BM Dredze M Could behavioral medicine lead the web data revolution? JAMA (2014) 311(14):1399 10.1001/jama.2014.1505 24577162 PMC4670613

[167] Burke LE Shiffman S Music E Styn MA Kriska A Smailagic A Ecological momentary assessment in behavioral research: addressing technological and human participant challenges. J Med Internet Res (2017) 19(3):e77. 10.2196/jmir.7138 28298264 PMC5371716

[168] Kaye J Curren L Anderson N Edwards K Fullerton SM Kanellopoulou N From patients to partners: participant-centric initiatives in biomedical research. Nat Rev Genet (2012) 13:371–6. 10.1038/nrg3218 22473380 PMC3806497

[169] DeFrancesco L To share is human. Nat Biotechnol (2015) 33(8):796–800. 10.1038/nbt.3309 26252130

[170] World Economic Forum. Unlocking the Value of Personal Data: From Collection to Usage. (2013). Available from: https://www.weforum.org/reports/unlocking-value-personal-data-collection-usage

[171] Hafen E Kossmann D Brand A Health data cooperatives – citizen empowerment. Methods Inf Med (2014) 53(2):1–5. 10.3414/ME13-02-0051 24514946

[172] Bresó A Sáez C Vicente J Larrinaga F Robles M García-Gómez JM. Knowledge-based personal health system to empower outpatients of diabetes mellitus by means of P4 medicine. Methods Mol Biol (2015) 1246:237–57. 10.1007/978-1-4939-1985-7_15 25417090

[173] Mozaffarian D Afshin A Benowitz NL Bittner V Daniels SR Franch HA Population approaches to improve diet, physical activity, and smoking habits: a scientific statement from the American Heart Association. Circulation (2012) 126(12):1514–63. 10.1161/CIR.0b013e318260a20b 22907934 PMC3881293

[174] Mozaffarian D Dietary and policy priorities for cardiovascular disease, diabetes, and obesity. Circulation (2016) 133(2):187–225. 10.1161/CIRCULATIONAHA.115.018585 26746178 PMC4814348

[175] Peek ME Ferguson MJ Roberson TP Chin MH. Putting theory into practice: a case study of diabetes-related behavioral change interventions on Chicago’s south side. Health Promot Pract (2014) 15(2 Suppl):40S–50S. 10.1177/1524839914532292 25359248 PMC4217132

[176] Goddu AP Roberson TS Raffel KE Chin MH Peek ME. Food Rx: a community-university partnership to prescribe healthy eating on the South Side of Chicago. J Prev Interv Community (2015) 43(2):148–62. 10.1080/10852352.2014.973251 25898221 PMC4416784

[177] Kahn R Davidson MB. The reality of type 2 diabetes prevention. Diabetes Care (2014) 37(4):943–9. 10.2337/dc13-1954 24652724 PMC3964495

[178] Vandenbroeck P Foresight: Tackling Obesities: Future Choices – Building the Obesity System Map. London: UK Gov Off Sci (2007).

[179] FAO, WFP, IFAD. The State of Food Insecurity in the World 2012. Rome (2012).

[180] Katan MB. Why the European Food Safety Authority was right to reject health claims for probiotics. Benef Microbes (2012) 3(2):85–9. 10.3920/BM2012.0008 22683835

[181] Mozaffarian D Ludwig DS Dietary guidelines in the 21st century – a time for food. JAMA (2010) 304(6):681–2. 10.1001/jama.2010.1116 20699461

[182] Debussche X. Is adherence a relevant issue in the self-management education of diabetes? A mixed narrative review. Diabetes Metab Syndr Obes (2014) 7:357–67. 10.2147/DMSO.S36369 25114578 PMC4122577

[183] Kousoulis AA Patelarou E Shea S Foss C Ruud Knutsen IA Todorova E Diabetes self-management arrangements in Europe: a realist review to facilitate a project implemented in six countries. BMC Health Serv Res (2014) 14(1):453. 10.1186/1472-6963-14-453 25278037 PMC4283086

